# Nutrient-Driven *O*-GlcNAcylation at Promoters Impacts Genome-Wide RNA Pol II Distribution

**DOI:** 10.3389/fendo.2018.00521

**Published:** 2018-09-10

**Authors:** Michael W. Krause, Dona C. Love, Salil K. Ghosh, Peng Wang, Sijung Yun, Tetsunari Fukushige, John A. Hanover

**Affiliations:** ^1^Laboratory of Molecular Biology, National Institute of Diabetes and Digestive and Kidney Diseases, National Institutes of Health, Bethesda, MD, United States; ^2^Laboratory of Cell and Molecular Biology, National Institute of Diabetes and Digestive and Kidney Diseases, National Institutes of Health, Bethesda, MD, United States

**Keywords:** *O*-GlcNAc, RNA-polymerase II, CTD, transcription, genetic, nutrients, polymerase dynamics, glycobiology

## Abstract

Nutrient-driven *O*-GlcNAcylation has been linked to epigenetic regulation of gene expression in metazoans. In *C. elegans, O*-GlcNAc marks the promoters of over 800 developmental, metabolic, and stress-related genes; these *O*-GlcNAc marked genes show a strong 5′, promoter-proximal bias in the distribution of RNA Polymerase II (Pol II). In response to starvation or feeding, the steady state distribution of *O*-GlcNAc at promoters remain nearly constant presumably due to dynamic cycling mediated by the transferase OGT-1 and the *O*-GlcNAcase OGA-1. However, in viable mutants lacking either of these enzymes of *O*-GlcNAc metabolism, the nutrient-responsive GlcNAcylation of promoters is dramatically altered. Blocked *O*-GlcNAc cycling leads to a striking nutrient-dependent accumulation of *O*-GlcNAc on RNA Pol II. *O*-GlcNAc cycling mutants also show an exaggerated, nutrient-responsive redistribution of promoter-proximal RNA Pol II isoforms and extensive transcriptional deregulation. Our findings suggest a complex interplay between the *O*-GlcNAc modification at promoters, the kinase-dependent “CTD-code,” and co-factors regulating RNA Pol II dynamics. Nutrient-responsive *O*-GlcNAc cycling may buffer the transcriptional apparatus from dramatic swings in nutrient availability by modulating promoter activity to meet metabolic and developmental needs.

## Introduction

Animals have evolved under conditions of fluctuating nutrient availability, requiring them to adapt in order to balance growth and survival. Thus, mechanisms exist to sustain essential cellular functions during prolonged starvation conditions while permitting a rapid metabolic response when conditions become replete. Transcriptional regulation is one such mechanism, controlling a subset of genes that are required for the acute catabolic or anabolic response to nutrient flux or changes in environmental stimuli.

Transcriptional regulation can occur at many different levels, including recruitment of RNA Polymerase II (Pol II) to the promoter and the transcription events of initiation, elongation, splicing, and termination. All of these activities are influenced by the differential phosphorylation of the C-terminal domain (CTD) of Pol II (1) and its associated factors. This “CTD-code” is subject to elaborate regulation by CTD-kinases (2, 3) and, together with other factors that stably or transiently associate with the polymerase complex, regulate gene expression (4–6).

Although recruitment of Pol II to the relevant promoters has long been thought to be the primary molecular control point for transcriptional responses, recent studies demonstrate that many stress and developmental genes have a pool of Pol II engaged at the promoter that is poised for rapid activation. This mode of control, referred to as Pol II promoter-proximal pausing, has emerged as a common means of transcriptional regulation among many, but not all, animals (7–13). From a biological perspective, this pool of promoter-associated polymerase has been suggested to serve a memory function, which may anticipate the need for rapid mobilization in response to environmental or developmental cues (14).

For the nematode *Caenorhabditis elegans*, life in the soil has subjected the animal to constant cycles of feast and famine throughout its evolution. *C. elegans* has adopted a number of developmental strategies to adapt to nutrient flux, including larval stage 1 (L1) arrest, dauer diapause, adult reproductive diapause and life extension in response to starvation (15–18). Many of these developmental decisions are under transcriptional control. In fact, it has been shown that starvation-induced L1 arrest in *C. elegans* is accompanied by enhanced Pol II promoter-proximal pausing, particularly at promoters for metabolic genes important for growth and stress responses (19, 20). However, unlike *Drosophila* and mammals, Pol II pausing does not appear to be a major mechanism of transcriptional regulation in *C. elegans* (21). Moreover, worms lack a NELF homolog (22), which is a major regulator of Pol II pausing found in other animals, raising the possibility that additional inputs might regulate Pol II dynamics in this organism.

Many different nutrient sensing pathways feed into transcriptional control and developmental decisions. We have previously demonstrated in *C. elegans* that dauer formation, the stress response, and adult longevity, are all strongly influenced by the Hexosamine Signaling Pathway (HSP) (23–25). For many key cellular targets, the HSP provides a dynamic, nutrient driven mechanism to influence protein function in response to the metabolic status of the cell (26, 27). The terminal step of the HSP is the transfer of O-linked *N*-acetylglucosamine (*O*-GlcNAc) to Ser and Thr amino acids in target substrates catalyzed by the enzyme *O*-GlcNAc transferase (OGT). The *O*-GlcNAc modification is reversed by the action of an *O*-GlcNAcase (OGA). This dynamic, post-translational, single sugar modification of nuclear and cytoplasmic proteins is reminiscent of phosphorylation and dephosphorylation of Ser/Thr residues by kinases and phosphatases. Thus, nutrient-driven *O*-GlcNAcylation is capable of producing extensive crosstalk with kinase-dependent, and other, signaling pathways influencing numerous aspects of cell physiology (26–29).

*O*-GlcNAc cycling has been genetically linked directly to the regulation of gene expression. We, and others, have shown that GlcNAcylation of chromatin-associated substrates is an evolutionarily conserved modification that is localized predominantly to the promoter regions of many genes, raising the possibility that promoter-proximal Pol II dynamics could be directly influenced by nutrient flux (30–33). RNA Pol II has been shown to be GlcNAcylated on the CTD (34–36), including on Ser-2 and Ser-5 residues of the CTD heptad repeat (37–39), and the *O*-GlcNAcase (OGA) shown to be part of the Pol II elongation complex interacting with Pol II pausing factors (40). Much of this previous work has been performed *in vitro* using recombinant OGT and OGA and examining the impact of these activities on template-driven transcription of model reporter constructs. The results of these studies suggest a model in which *O*-GlcNAc plays a role in preinitiation complex formation and interplays with the “CTD code” of phosphorylation on RNA Pol II (34–39).

In efforts to determine the function *O*-GlcNAc on transcription in an intact organism, we have analyzed deletion alleles encoding these enzymes in the mouse and in *Drosophila*. In mouse, OGT is essential (41–43), but OGA null alleles survive until the perinatal period, directly demonstrating that *O*-GlcNAcase is not essential for transcription and development (44). Loss of OGA in *Drosophila* is also non-essential, but impacts the epigenetic machinery allowing *O*-GlcNAc accumulation on RNA Polymerase II and numerous chromatin factors including TRX, ASH1, and SET (33). Here, we have extended the analysis to viable knockout alleles in *C. elegans* of the genes encoding OGT and OGA to explore the role of *O*-GlcNAcylation on Pol II distribution and transcription in a whole organism.

Chromatin immunoprecipitation (ChIP) of starved or fed samples with multiple antibodies specific for either the *O*-GlcNAc modification itself or Pol II CTD phosphorylated isoforms revealed a near constant distribution of Pol II across most genes in wild type animals regardless of growth conditions. In contrast, loss of OGT-1 activity resulted in dramatic changes to Pol II distribution in response to nutrient flux, particularly for genes associated with high levels of GlcNAcylated chromatin at their promoters; a similar, but less dramatic effect was seen with loss of OGA-1 activity. We also find that animals lacking OGT, and to a lesser extent OGA, activity have substantially altered gene expression responses in starved and fed conditions compared to wild type animals. Our results demonstrate that dynamic GlcNAcylation of Pol II and other chromatin associated substrates, predominantly localized at promoters, is part of a homeostatic mechanism that ensures a constant steady state Pol II distribution for most genes during fluctuating nutritional conditions. The post-translational modification of chromatin-associated substrates by nutrient responsive GlcNAcylation provides a direct link between the cellular metabolic state and Pol II promoter-proximal dynamics with epigenetic implications for the organism.

## Results

### Promoter-associated *O*-GlcNAc levels are maintained by *O*-GlcNAc cycling in conditions of both starvation and nutrient excess

Previously, we showed that *O*-GlcNAcylated substrates reside at the promoter regions of many genes, microRNAs, and non-coding RNAs in *C. elegans* (32). Transcriptional changes resulting from the disruption of *O*-GlcNAc cycling described previously (32) suggested that GlcNAcylation might directly impact transcription by modifying one or more chromatin-associated substrates in response to nutrient flux. To extend these findings, we have used the viable null alleles of *O-*GlcNAc to determine the impact of loss of *O*-GlcNAc cycling on RNA polymerase behavior in response to changes in nutrient availability.

Antibodies that specifically recognize the *O*-GlcNAc epitope were used in chromatin immunoprecipitation (ChIP) experiments with synchronous, first larval stage (L1) wild type animals under starved and fed conditions [Experimental Procedures; (32)]. Chromatin derived probes were used for whole genome tiling array hybridization (ChIP-chip) and the data normalized to a common input chromatin sample. We have found that our ChIP-chip data provided a less biased, hybridization-based signal across genes compared with new technology, such as ChIP-seq, and allows direct comparisons with previous work, including our own studies in *Drosophila melanogaster* (33) and *C. elegans* (32). To allow visualization of the ChIP-chip data from multiple genes simultaneously, a common gene model (metagene) of uniform length was defined that extended from the translational start to stop codons based on genome version WS195 to match the arrays that were used (Experimental Procedures); our analyses also included 3 kb upstream of the start site corresponding to hightened *O*-GlcNAc signal intensity.

To validate our ChIP-chip data, multiple chromatin preparations and immunoprecipitations with many different antibodies to *O*-GlcNAc and Pol II were used in various combinations to demonstrate consistency and reproducibility of the results (Experimental Procedures). As previously reported, almost all Pol II antibodies gave similar patterns across the genome regardless of their advertised specificity or preference for different Pol II CTD phosphorylated isoforms (19)(Supplemental Figure 1). These results are consistent with our own assays of specificity using both peptide dot blots and *in vivo* antibody staining in early embryos (Supplemental Figures 1, 2). Our data sets also replicated and confirmed a previous study that identified a set of genes with a high level of promoter-proximal Pol II compared to the gene bodies (19)(data not shown). Therefore, we had high confidence that this data could be used to examine chromatin distributions among various gene classes and multiple genetic mutants to explore possible correlations that would inform the functions of chromatin *O*-GlcNAcylation, if any.

**Figure 1:**
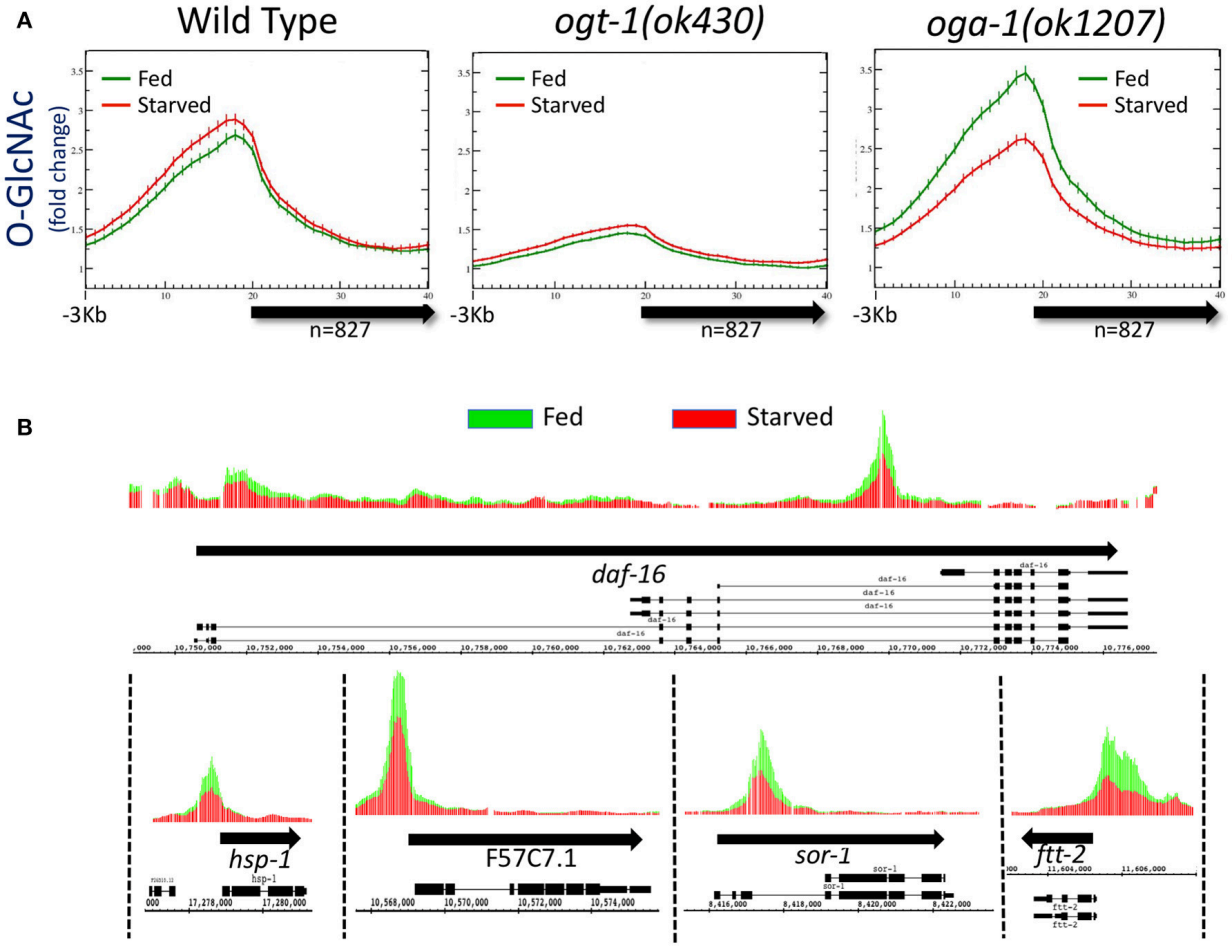
Levels of promoter region *O*-GlcNAcylation are actively regulated in wild type animals. **(A)** Chromatin-associated *O*-GlcNAc signals, as determined by ChIP-chip, are shown relative to a unitary, metagene model (bold arrow) along with 3 kb flanking sequence upstream of the gene model. Fold enrichment over the mean chip value is shown on the y-axis; the x-axis indicates the bin number for consolidated data. Data shown is averaged from 827 previously identified *O*-GlcNAc “marked” genes in wild type or *O*-GlcNAc cycling mutants (*ogt-1(ok430)* and *oga-1(ok1207)*) (32) for both starved (red) and fed (green) conditions; standard error is indicated for each point on the plots. There was little to no change in promoter region *O*-GlcNAcylation in wild type worms in response to changes in nutrient conditions. Comparing the ratio of the promoter region peak to gene body nadir values (Starved = 2.30 ± 0.09; Fed = 2.20 ± 0.08) showed no statistical difference (*p* = 0.397) for wild type animals. The promoter region enrichment observed for *ogt-1* mutant animals represents background as these animals lack the ability to *O*-GlcNAcylate substrates, providing a useful genetic control for ChIP. The dramatic up-regulation of promoter region *O*-GlcNAcylation in response to feeding in *oga-1* mutants reflects disruption of the dynamic cycling of *O*-GlcNAc on chromatin-associated substrates. Comparison of the ratios for promoter region peak to gene body nadir values (Starved = 2.11 ± 0.08; Fed = 2.62 ± 0.11) is significantly different (*p* = 0.0002) in fed vs. starved conditions in *oga-1* mutants. **(B)** The *oga-1(ok1207) O*-GlcNAc ChIP-chip profiles for several individual genes, as indicated, in both starved (red) and fed (green) conditions are shown. In each case, the promoter region *O*-GlcNAcylation appears to be nutrient responsive, rising dramatically for fed conditions in the *oga-1* mutant background. Thick black arrows indicate the transcribed region with the exon/intron gene structure for one or more previously defined gene products illustrated below.

We first examined the list of 827 genes previously identified as being associated with elevated promoter *O*-GlcNAc marks in *C. elegans* (32); note that one gene model on the original list is no longer valid, resulting in 826 genes, but we have retained the original naming convention for clarity. Comparison of wild type starved vs. fed data for these “marked” genes revealed nearly identical profiles of *O*-GlcNAc signal relative to the common gene model with a strong promoter region bias in distribution (Figure 1A); this was true for each of two independent anti-*O*-GlcNAc antibodies (RL2 and HGAC85) assayed by whole genome ChIP (data not shown). The constant distribution of *O*-GlcNAc in response to nutrient flux was surprising since total *O*-GlcNAc on cellular proteins increases in response to nutritional flux in worms (45). An analysis of the genomic sequence associated with the *O*-GlcNAc promoter intervals revealed an enrichment of two repetitive sequence motifs: GAGAGAGA, ACACACAC, and their inverse complements (Supplemental Figure 3). These simple repeats have also been noted in promoter regions in other transcription factor ChIP studies (46, 47) suggesting their presence may simply reflect a general property of promoters rather than a binding site preference for GlcNAcylated chromatin-associated proteins *per se*. The constant *O*-GlcNAcylation at promoters in response to nutrient flux suggested that *O*-GlcNAc levels might be actively regulated at these sites in wild type animals.

### Promoter GlcNAcylation responds aberrantly to nutrient flux when *O*-GlcNAc cycling is blocked

To determine if disruption of active *O*-GlcNAc cycling had an effect on the distribution of chromatin GlcNAcylation in starved vs. fed animals, we assayed the same 827 marked genes in mutants in which either OGT-1 or OGA-1 activity had been eliminated by genomic deletions. We have previously shown that the *ogt-1(ok430)* mutants lack *O*-GlcNAc transferase activity (23) and we used this enzymatic null allele to threshold ChIP-chip experiments in starved animals to define the list of 827 *O*-GlcNAc marked genes (32). As expected, the *O*-GlcNAc ChIP signals in fed or starved *ogt-1(ok430)* mutants is quite low and similar to that observed and defined as background [Figure 1A; (32)]. The observed over representation of promoter regions in this mutant, which lacks the enzymatic activity needed to generate the epitope being ChIPed, likely reflects the relative open chromatin associated with promoters that is commonly observed in ChIP studies (48–50).

We have also characterized a viable null mutant in the *O*-GlcNAcase gene in *C. elegans* (*oga-1(ok1207)*) (24). ChIP data from *oga-1(ok1207)* mutants that cannot reverse the *O*-GlcNAc modifications on substrates revealed changes in response to the nutrient status of the organism (Figure 1A). Promoter regions of the 827 *O*-GlcNAc marked genes showed a large and statistically different (*p* = 0.0002) increase in *O*-GlcNAcylation signals in response to feeding, an effect that could also be easily visualized on representative individual gene promoters (Figure 1B). The *oga-1(ok1207)* results demonstrated that chromatin-associated, GlcNAcylated substrates are rendered nutrient-sensitive when *O*-GlcNAc cycling is disrupted. Taken together, these findings suggest that in wild type animals the dynamic cycling of *O*-GlcNAc is required to maintain the near constant, or buffered, levels of *O*-GlcNAcylation observed at gene promoters.

As described above, the constant distribution of promoter-associated *O*-GlcNAc in response to nutrient flux was surprising because total protein *O*-GlcNAcylation increased in *C. elegans* in response to nutritional flux in the form of excessive glucose (45). We re-examined total protein *O*-GlcNAcylation in L1 populations for wild type, *ogt-1(ok430), ogt-1(ok1474), oga-1(ok1207)* mutant animals by Western blots, comparing starved vs. fed conditions that mimicked those used for our ChIP studies. Although *O*-GlcNAc signals were either not detected (*ogt-1* mutants) or too low (wild type) to conclude any effect in these experiments, the *oga-1(ok1207)* animals clearly demonstrated dramatically elevated levels of protein *O*-GlcNAcylation that were further increased in fed conditions when compared to starved (Supplemental Figure 4). Thus, nutritional status can drive excessive levels of protein *O*-GlcNAcylation with the potential to affect promoter-associated substrates.

### RNA polymerase II is dynamically *O*-GlcNAcylated *in vivo*

The C-terminal domain (CTD) of mammalian RNA Polymerase II (Pol II) has been reported to be GlcNAcylated on Ser and Thr residues of the canonical heptad repeat sequences (YSPTSPS) and closely related variants (34, 35, 51). The *C. elegans* large subunit of Pol II, encoded by the *ama-1* gene, has 37 heptad repeats within its CTD of which 10 match the canonical sequence exactly.

**Figure 2:**
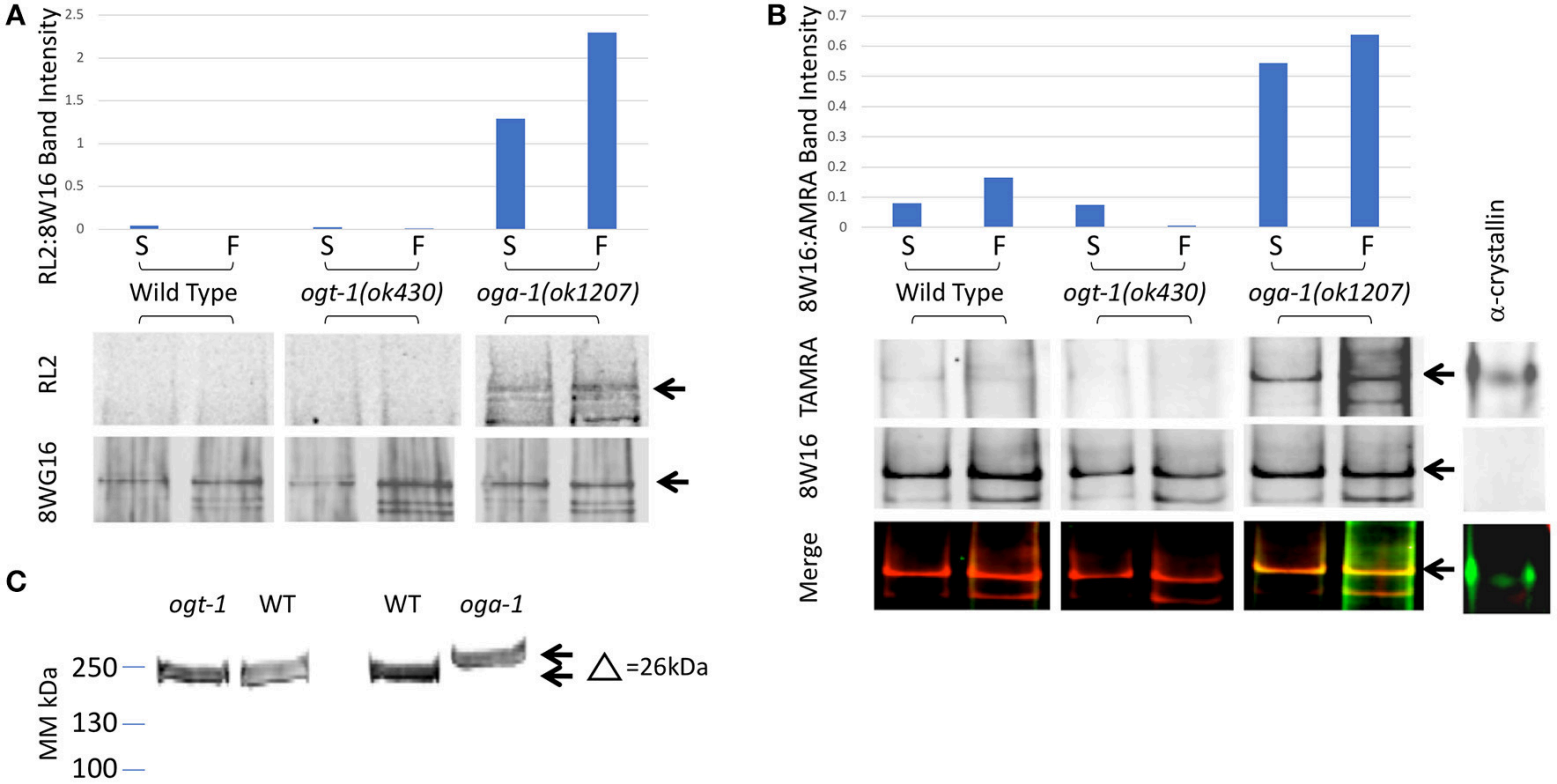
*C. elegans* RNA Polymerase II is *O*-GlcNAcylated. **(A)** RNA Polymerase II (Pol II) was immunopurified from extracts prepared from starved (S) and fed (F) L1 larvae using wild type or *O*-GlcNAc cycling mutant strains. Western blots were probed with an anti-*O*-GlcNAc antibody (RL2) or anti-Pol II antibody that recognizes isoforms independent of CTD phosphoepitopes (8WG16), as indicated. The position of the major Pol II band is indicated by arrows in the blots. This band was quantified in each lane using ImageJ and the relative ratio of RL2 to 8WG16 signal intensity plotted in the graph above. The *oga-1* mutant strain has dramatically increased levels of *O*-GlcNAcylated Pol II that is further increased upon feeding. **(B)** RNA Pol II was immunopurified from extracts prepared from starved (S) and fed (F) animals as in **(A)**. A modified recombinant galactosyltransferase was used to introduce a GalNAz label to *O*-GlcNAc modified proteins (52). The GalNAz label was reacted with an alkyne-TAMRA probe using “Click” chemistry (53). The top row of Western blot images shows the anti-TAMRA antibody detection results. As a positive control, α-crystallin (20 pg) was detected using the same method. The second row of images shows the anti-Pol II signal (8WG16) from an identical blot; note the absence of signal for α-crystallin. The last row of images shows the overlay of signals from the two fluorophores corresponding to *O*-GlcNAc and Pol II. The position of the major Pol II band is indicated by arrows in all three blot images. This band was quantified in each lane using ImageJ and the relative ratio of TAMRA to 8WG16 signal intensity plotted in the graph above. **(C)** Pol II in *oga-1* mutants has a higher molecular mass. The post-nuclear supernatants (see Experimental Procedures) from wild type and mutant strains were run on 10–20% SDS-PAGE gels and transferred for immunoblotting as described in Experimental Procedures. The primary antibody was the RNA Pol II Ser-2-P antibody ab5095 and the secondary IR-labeled anti-mouse IgG. The blot was scanned with an Oddysey IR scanner as described previously (25).

We immunoprecipitated Pol II from starved and fed wild type and mutant L1 stage animal extracts using a Pol II antibody (8WG16) that recognizes phosphorylated and non-phosphorylated isoforms (19, 48) (Experimental Procedures). Immunoprecipitation was followed by detection of *O*-GlcNAc by two independent methods. Using antibodies specific for *O*-GlcNAc, we were able to detect Pol II in the *oga-1 (ok1207)* mutant where *O*-GlcNAc removal is blocked; little or no GlcNAcylated Pol II was detected in wild type extracts and none was ever detected in the *ogt-1(ok430)* mutant (Figure 2A). We next used a highly sensitive chemoenzymatic detection method (52) based on the selectivity of galactosyltransferase to modify terminal *O*-GlcNAc (54, 55). This method uses ‘Click’ chemistry to place a fluorescent tag (TAMRA) onto *O*-GlcNAc-modified proteins that can subsequently be identified with an anti-TAMRA antibody on immunoblots. As shown in Figure 2B, Pol II GlcNAcylation was readily detected in both the *O*-GlcNAcase mutant strain [*oga-1(ok1207)*] and, to a lesser extent, in wild type; GlcNAcylation of Pol II was undetectable in the *ogt-1(ok430)* strain. When the same blots were probed with an anti-Pol II antibody (8WG16), all stains were shown to have nearly identical levels of Pol II. There was a readily detectable increase in Pol II GlcNAcylation in fed vs. starved samples in the *oga-1(ok1207)* mutant, reminiscent of the fed response observed by *O*-GlcNAc ChIP (Figure 2A). In addition, the migration of intact Pol II was detectably different in comparing the three strains [N2, *ogt-1(ok430)* and *oga-1(ok1207)*] using Pol II antibodies, with an upward shift in migration of approximately 26 kDa (Figure 2C), suggesting that Pol II is highly modified in the *oga-1(ok1207)* strain that lacks *O*-GlcNAcase. Thus, only a small fraction of total Pol II is likely to be GlcNAcylated at steady state due, in large part, to active cycling by both OGT-1 and OGA-1. When *oga-1* is deleted, the levels of modified Pol II increase greatly. The relatively small fraction of GlcNAcylated Pol II and extensive cross-linking required for ChIP precluded us from doing sequential IP studies to determine if the promoter region *O*-GlcNAc signal is predominantly due to GlcNAcylated Pol II. Therefore, we took advantage of the many tools available to examine Pol II dynamics to determine if altered GlcNAcylation altered Pol II behavior.

#### Genome-wide analysis of RNA pol II isoforms in *C. elegans* L1 larvae

Since Pol II was a confirmed nutrient-driven target of GlcNAcylation in the worm, we have directly examined the genome-wide behavior of Pol II using the genetic and biochemical tools at our disposal. This analysis was carried out at the whole-genome level, allowing a robust metagene analysis that would not be possible by examination of smaller data sets or by examining single genes. Over 36 whole genome arrays were analyzed in this way, with representative data presented for simplicity.

The CTD of Pol II in animals and yeast is regulated by phosphorylation of the heptad repeat (YSPTSPS) on Ser-2,−5, and−7; Ser-5-P is associated with initiating Pol II, whereas increasing Ser-2-P is linked to elongation [reviewed in (3)]. To determine the genome-wide distribution of Pol II in L1s, we carried out ChIP-chip experiments using anti-Pol II antibodies that had different specificities with respect to CTD post-translational modifications. Antibody 8WG16 is considered phospho-independent, recognizing both phosphorylated and non-phosphorylated forms of the CTD and has been used previously in *C. elegans* for ChIP by multiple groups (19, 20, 48, 56). Antibody 5095 (Abcam) recognizes predominantly Pol II phosphorylated on Ser-2 within the CTD. We also used two non-commercial anti-Pol II antibodies raised against di-heptad CTD repeat peptides phosphorylated on either the Ser-5 or Ser-2 positions (57, 58). We confirmed the specificity of each of these phospho-specific Pol II antibodies using immunoblot dot blots (Supplemental Figure 1B) as well as *in vivo* staining of *C. elegans* embryos that have characteristic germline vs. somatic cell nuclear staining patterns for CTD Ser-2-P- or Ser-5-P-specific antibodies (22, 59, 60)(Supplemental Figure 2). As others have recently reported (19, 20, 56), we found that the ChIP profiles for all of these Pol II antibodies were very similar (Supplemental Figures 1A, 5). From these extensive genome-wide analyses, we concluded that for *C. elegans* L1 chromatin, Pol II ChIP patterns were very reproducible using a variety of antibodies and multiple, independent chromatin preparations. Moreover, it was not possible to discriminate between initiating or elongating forms of Pol II based on *in vivo* ChIP with these antibodies despite their clear difference for CTD phospho-isoforms *in vitro*.

### *O*-GlcNAc marked promoters show enhanced promoter proximal pol II with a distribution that is buffered from nutrient flux in wild type animals

**Figure 3:**
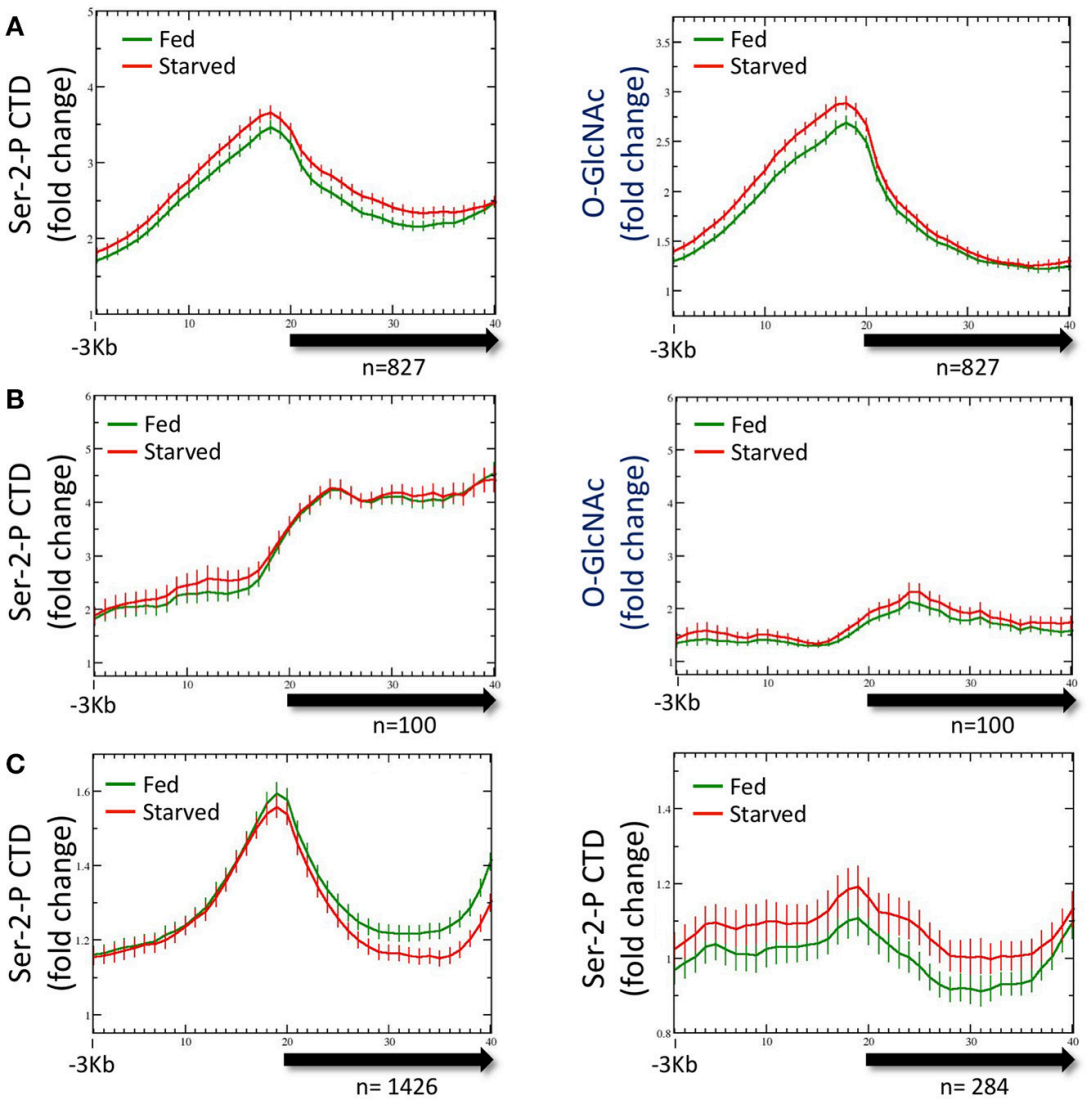
RNA Polymerase II profiles for *O*-GlcNAc marked genes are similar in both starved and fed conditions. **(A)** RNA Pol II Ser-2-P (ab5095), representative of multiple Pol II antibodies used, and *O*-GlcNAc ChIP profiles for the 827 *O*-GlcNAc marked gene set with axis and gene model displays as described in Figure 1; the *O*-GlcNAc profile is duplicated from Figure 1A (right panel) for side by side comparison to the corresponding Pol II data. Very little difference was observed for the Pol II distribution in comparing fed and starved conditions. Note that while Pol II and *O*-GlcNAc profiles are similar in the promoter regions, the Pol II signal extends into the gene body whereas *O*-GlcNAc does not. **(B)** To determine if the *O*-GlcNAc marked gene set behavior was unusual in being unchanged regardless of feeding condition, we examine several gene sets that were selected using different criteria. Show is a set of 100 genes that had high levels of RNA Pol II within the gene body, but low promoter region *O*-GlcNAc signals. This gene set also shows nearly identical profiles for Pol II (left panel) in either starved or fed conditions, lacks a strong signal for promoter region *O*-GlcNAc (right panel), and has an overall profile that is quite distinct from the 827 *O*-GlcNAc marked genes. **(C)** Genes with nutritionally responsive expression have corresponding changes in Pol II occupancy. As many gene sets showed similar profiles for Pol II in both starved and fed conditions, we identified genes that were nutritionally responsive to these two conditions based only on changes in gene expression (log_2_ fold change greater than or equal to 1.5 when comparing starved and fed values). The left panel shows the Pol II profile, represented by the Ser-2-P data, for 1426 genes that are up-regulated in fed conditions compared to starved values. As expected, the fed Pol II profile increases in the gene body of these genes. For genes that are up-regulated in starved conditions (*n* = 284; right panel), the starved Pol II profile is greater than the fed. Thus, the Pol II ChIP profile changes are consistent with the gene expression data for those gene showing the most dynamic response to feeding condition whereas Pol II occupancy profiles for nutritionally unresponsive genes are correspondingly homeostatic.

As noted above, both Pol II and total promoter region *O*-GlcNAc levels are maintained in wild type animals for either starved or fed conditions by dynamic *O*-GlcNAc cycling. To determine if the distribution of Pol II changed in response to nutrient flux, data from ChIP experiments with multiple Pol II antibodies (Experimental Procedures) were analyzed with respect to both starved and fed conditions. Regardless of the Pol II antibody used for ChIP, we found substantial levels of Pol II at the promoter of the set of 827 genes defined as being GlcNAcylated, as represented by the Ser-2-P antibody profile (Figure 3A). Like *O*-GlcNAc signals, the Pol II profiles were very similar in both starved and fed conditions, with differences in 5′ promoter peak to gene body nadir ratios not reaching statistical difference (*p* = 0.681). We noted that the Pol II distribution within the 5′ promoter region was very similar to the GlcNAcylated chromatin profile, whereas a very distinct pattern exists within the gene body as modeled by the metagene (Figure 3A). Thus, in wild type animals neither the profile nor magnitude of Pol II distribution for the 827 *O*-GlcNAc marked genes was significantly altered in response to nutrient flux.

To determine if the averaged profile of Pol II for most genes remained unchanged during nutrient flux in wild type animals, we analyzed the Pol II ChIP data for gene sets selected for different characteristics. The list of genes used for this analysis are given in Supplemental Table 1. For example, data from a set of 100 genes selected for high levels of Pol II at the promoter and in the gene body gave a very different pattern, but similarly showed no change between starved and fed conditions (Figure 3B); these gene promoters had relatively low levels of *O*-GlcNAcylation. We also examined the Pol II profiles for 1426 genes transcriptionally upregulated (log_2_ fold change of 1.5 or greater) in fed conditions compared to starved and 284 genes upregulated in starved compared to fed (Figure 3C). For both of these gene sets, the condition for which they were transcriptionally upregulated corresponded to increased Pol II signals across the gene body, although starved and fed condition profiles overall were not significantly different. We concluded that in wild type animals, (1) nutrient flux does not affect the steady state level of Pol II occupancy for most genes, including those defined as *O*-GlcNAc marked, (2) high levels of promoter region *O*-GlcNAcylation were strongly correlated with a group of genes exhibiting a strong 5′ promoter bias in the Pol II profile, (3) Pol II profiles are different and concordant for sets of genes that show dramatic changes in expression, and (4) Pol II profiles reflect the criteria used to select the gene set.

**Figure 4:**
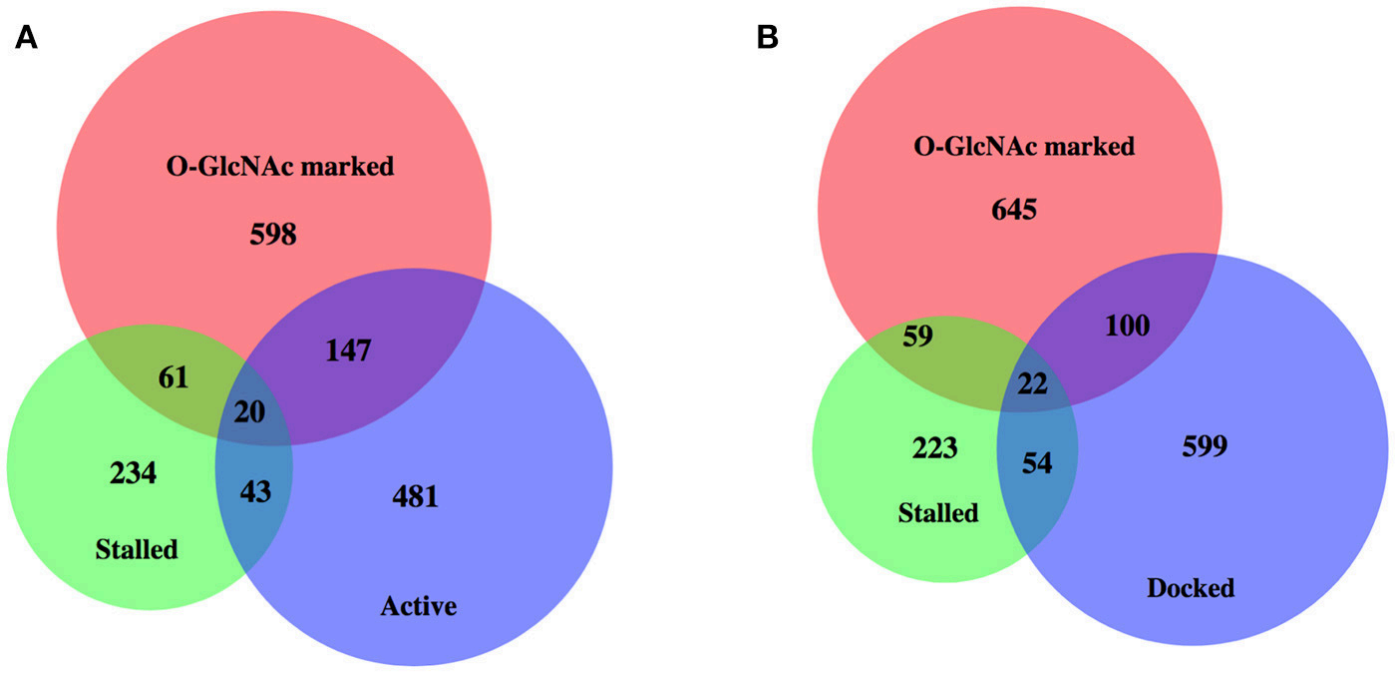
High levels of promoter Pol II for most *O*-GlcNAc marked genes do not indicate polymerase pausing. Proportional area Venn diagrams of gene set overlap among the 827 *O*-GlcNAc marked genes and other gene sets defined as having paused polymerase. Overlaps between the 827 *O*-GlcNAc marked genes (red) and genes identified as having promoter proximal paused Pol II in starved L1s from Zhong et al. (47) (*n* = 358, green) and active [*n* = 691, blue; **(A)**] or docked [*n* = 775, blue; **(B)**] as defined by Maxwell et al. (20). The majority of *O*-GlcNAc marked genes are not included in any of these gene sets previously identified as having promoter proximal Pol II in starved conditions in wild type animals.

Enhanced promoter proximal Pol II accumulation, or pausing, has previously been reported in *C. elegans* by multiple groups under starved conditions (19, 47). We compared each of these gene sets with our 827 *O*-GlcNAc marked genes, all of which had high levels of promoter proximal Pol II signals (Figure 4); gene lists are available in Supplemental Table 1. Although there was some overlap, these defined gene sets were much more different from each other than they were similar. In particular, most of the *O*-GlcNAc marked genes were not among those genes that have more rigorously been defined as having promoter proximal pausing of Pol II, suggesting a more general role for *O*-GlcNAcylation in Pol II dynamics.

### *O*-GlcNAc cycling is required to buffer pol II occupancy and distribution from changes in nutrient flux

**Figure 5:**
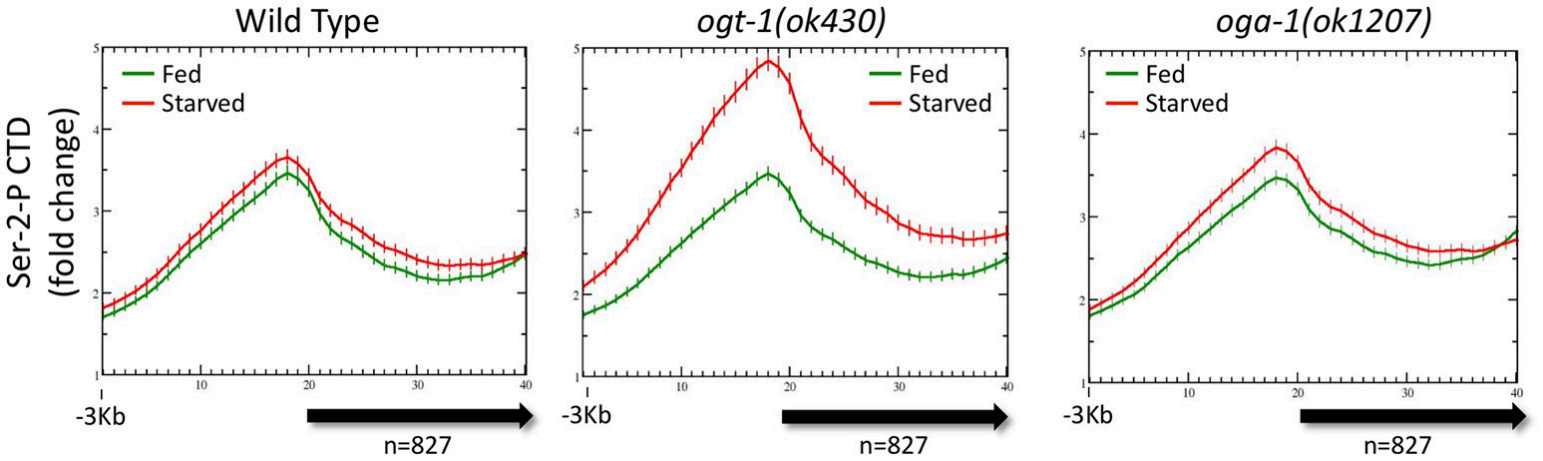
Pol II profiles become deregulated in response to nutrient flux in animals lacking active *O*-GlcNAcylation. The CTD Ser-2-P Pol II profile (ab5095), representative of all Poll II antibodies tested, for the 827 *O*-GlcNAc marked genes are shown relative to the metagene model, as described in Figure 1. The ChIP data from wild type (**left**; duplicated for comparison from Figure 3A, left) or *O*-GlcNAc cycling mutants *ogt-1(ok430)* (**center**) and *oga-1(ok1207)* (**right**) L1 animals is shown under either starved (red) and fed (green) conditions. Whereas wild type and *oga-1(ok1207)* animals showed no statistically significant change in Pol II profiles for this set of genes in response to nutrient flux, loss of OGT activity resulted in dramatic changes in the 5′ promoter region to gene body nadir signal ratios (Starved = 1.81 ± 0.08; Fed = 1.57 ± 0.05), with starved conditions having significantly (*p* = 0.008) more Pol II occupancy across the promoter and gene body.

It was possible that there were differences in the genome-wide distribution of Pol II in the *O*-GlcNAc cycling mutants, mirroring difference in the levels of Pol II GlcNAcylation we observed by Western blot analysis. Chromatin from *ogt-1* or *oga-1* mutant populations (starved or fed) were ChIPed with each of the four anti-Pol II antibodies described above. The *ogt-1(ok430)* mutant results were particularly dramatic, with very different starved vs. fed profiles and significant differences (*p* = 0.008) in the 5′ promoter to gene body nadir values (Figure 5). In contrast, the *oga-1(ok1207)* mutant profile and peak ratios were very similar to wild type animals and not significantly different (*p* = 0.478). We also examined the behavior of Pol II in the *O*-GlcNAc mutants for several other groups of genes referenced above using the four anti-Pol II antibodies (data not shown). In each case, the results were similar to the effects shown in Figure 5, but with a diminished magnitude in deviation from wild type that was correlated with the relative abundance of *O*-GlcNAc at the promoter region. Taken altogether, the mutant studies demonstrate that disruption of *O*-GlcNAcylation results in deregulation of homeostatic mechanisms that normally function to maintain the steady state Pol II distribution for many genes during periods of nutrient flux.

### Promoters with dynamic changes in GlcNAcylation are dysregulated for pol II occupancy in response to nutrient flux

**Figure 6:**
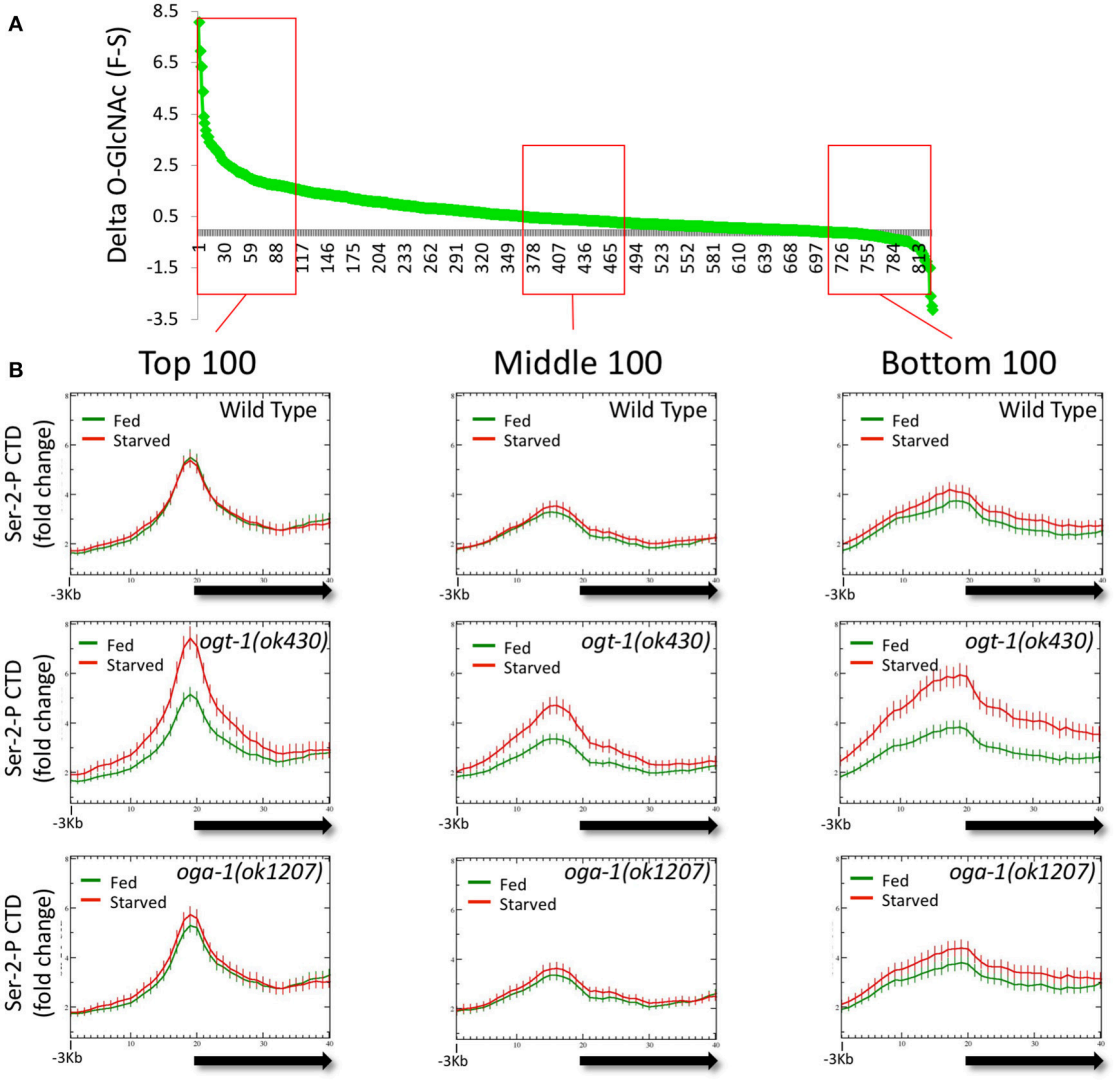
Loss of *O*-GlcNAc activity alone, and not nutrient responsive *O*-GlcNAcylation at promoters, correlates with Pol II dysregulation. The difference in ChIP *O*-GlcNAc signal values for each of the 827 *O*-GlcNAc marked genes in starved vs. fed conditions (delta-*O*-GlcNAc) was calculated from *oga-1(ok1207)* mutant data and plotted in rank order **(A)**. Three bins of 100 genes each were selected from the top, middle, or bottom rankings and the CTD Ser-2-P Pol II ChIP profile (ab5095), representative of all Poll II antibodies tested, was plotted (as described in Figure 1) for wild type and the *O*-GlcNAc cycling mutants **(B)**. Regardless of ranking based on the nutrient responsiveness of *O*-GlcNAcylation, wild type, and *oga-1* mutants showed very little difference in starved vs. fed Pol II profiles. In contrast, *ogt-1* mutants lacking *O*-GlcNAc activity, show dramatic differences in Pol II profiles in starved vs. fed conditions regardless of nutrient responsive ranking. Gene lists are available in Supplemental Table 1.

The observation that dramatic changes in the *O*-GlcNAc ChIP signals at promoters in the *O*-GlcNAc cycling mutants were associated with striking deregulation of Pol II occupancy (Figure 5) prompted us to examine if these two properties were linked. We reasoned that by looking at promoters that accumulated the highest levels of *O*-GlcNAc upon blocking *O*-GlcNAc cycling, we might enrich for those genes with dramatically altered Pol II distributions. We calculated the difference in *O*-GlcNAc signal between fed and starved samples (delta *O*-GlcNAc) in *oga-1(ok1207) O*-GlcNAcase mutants for the 827 marked promoters and plotted those in rank order (Figure 6A). One hundred genes from each of three different regions of the distribution representing the highest, middle, and lowest delta *O*-GlcNAc values were chosen and the distribution of Pol II CTD Ser-2-P was examined in either starved or fed conditions for wild type and mutant chromatin. As shown in Figure 6B, each gene set had a distinctive Pol II profile, with the highest levels of Pol II observed in the genes with the greatest change in *O*-GlcNAc when comparing starved and fed animal. More importantly, while wild type and *oga-1(ok1207)* mutants showed very similar results for all gene sets, the *ogt-1(ok430)* mutants show dramatic difference in starved vs. fed Pol II profiles. Finally, we noted that when the *O*-GlcNAc accumulation was highest in fed samples (e.g., high delta *O*-GlcNAc value), the 5′ Pol II CTD Ser-2-P occupancy was highest in starved samples in *ogt-1(ok430)* mutants. Therefore, independent of the magnitude of change in promoter region GlcNAcylation during starved or fed conditions, Pol II profiles remained remarkably buffered against nutrient flux in wild type and *oga-1* mutants. In contrast, *ogt-1* mutants have lost that buffering capacity and have dramatically increased levels of Pol II specifically in starved conditions. The dysregulation of Pol II occupancy in *ogt-1*, but not *oga-1*, mutants demonstrates that *O*-GlcNAcylation activity alone has a major role in buffering polymerase occupancy against nutrient flux. Once *O*-GlcNAcylated, the additional levels of this modification resulting from loss of *O*-GlcNAcase activity does little to influence Pol II occupancy.

### *O*-GlcNAc cycling mutants have an altered transcriptome and respond differentially to nutritional status

**Figure 7:**
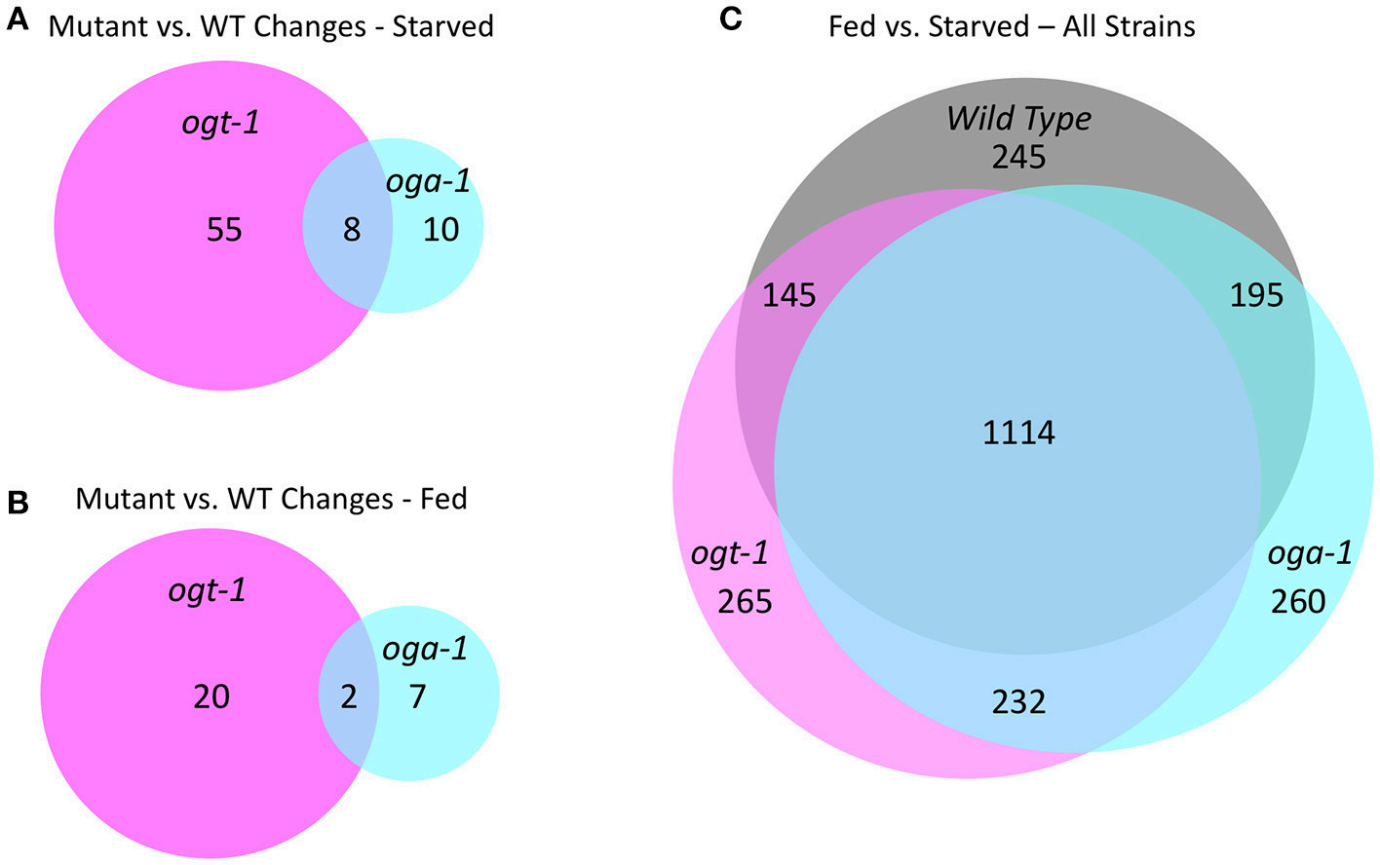
*O*-GlcNAc cycling mutants exhibit transcriptional changes in response to nutritional status. **(A)** An area proportional Venn diagram showing the genes deregulated greater than 2.8-fold (log_2_ = 1.5) in starved L1 larvae in *ogt-1 (ok430)* and *oga-1(1207)* mutant strains compared to wild type (WT) animals. **(B)** An area proportional Venn diagram showing the genes deregulated 2.8-fold or greater in fed L1 larvae in *ogt-1 (ok430)* and *oga-1(ok1207)* mutant strains compared to wild type worms. **(C)** An area proportional Venn diagram showing the uniquely deregulated genes comparing starved and fed worms in wild type, *ogt-1 (ok430)* and *oga-1(ok1207)* mutant strains. Gene lists for each condition and genotype can be found in Supplemental Table 2. All transcriptional data are available in GEO accession number GSE18132.

We previously reported that growth arrested L1 larvae harboring null alleles of *ogt-1* and *oga-1* have dramatically altered gene expression compared to wild type animals (roughly 700 and 500 deregulated genes, respectively) (32). In this report, we extended this analysis to fed L1 larvae and compared wild type, *ogt-1(ok430)*, and *oga-1(ok1207)* mutants; the entire dataset is available as GEO accession number GSE18132. We limited our bioinformatic analysis here to those genes substantially deregulated with a 2.8-fold (log_2_ = 1.5) or greater change in gene expression. As shown in Figure 7A, starved L1s have 63 genes that were differentially deregulated in *ogt-1(ok430)* compared to wild type animals, whereas *oga-1(ok1207)* had 18 differentially deregulated genes. As noted before (32), the genes deregulated in *ogt-1* are associated with innate immunity and the stress response. Of particular interest is the enhanced expression of glutathione transferases (Supplemental Table 2). For *oga-1*, the 18 deregulated genes were enriched in serpentine receptors involved in chemosensation, and C-type lectins. We next examined the changes in gene expression for fed L1s (Figure 7B). Here, 22 genes were at least 2.8-fold different in *ogt-1(ok430)*; these genes were bioinformatically enriched in membrane receptors involved in chemosensation (Supplemental Table 2) and included the piwi-like protein *prg-1* that was highly down-regulated in the fed conditions compared to wild type. The *prg-1* gene encodes an argonaut protein involved in regulating piRNAs, microRNAs and select protein coding mRNAs (61). Finally, genes deregulated in *oga-1(ok1207)* fed L1 animals were similar to that of starved larvae; serpentine and G-coupled receptors. Thus both in the fed and starved conditions, the *ogt-1* mutants exhibited more striking deregulation of gene expression than the *oga-1* mutants in comparisions to wild type.

To examine the overall nutrient response of the *O*-GlcNAc cycling mutants from a different perspective, we examined the transcriptional responses of three strains in both conditions (Figure 7C; Supplemental Table 1), again applying a high threshold of 2.8-fold change (log_2_ = 1.5) for the analysis. Although there was a core group representing ~85% of the genes that were commonly deregulated in response to nutrient status, each of the O*-*GlcNAc cycling mutants showed a substantially altered and strain-specific transcriptional response (Figure 7C). Wild type L1s had 245 nutrient responsive genes that were not detected in either *ogt-1(ok430)* or *oga-1(ok1207)* mutants in response to feeding. The *ogt-1(ok430)* strain had 265 genes uniquely deregulated in response to feeding while *oga-1(ok1207)* had 260 deregulated genes. This is consistent with the changes in polymerase occupancy we have observed and the transcriptional alterations seen in *ogt-1* and *oga-1* mutants compared to wildtype. Finally, we examined the relationship between the most nutrient responsive genes in all three strains to our 827 *O*-GlcNAc marked gene set (Supplemental Figure 6). In all pair-wise comparisions, the overlap failed to meet statistical significance (Fisher's Exact Test, *p* < 0.05) in exceeding random chance.

## Discussion

### *O*-GlcNAc cycling is a nutrient-responsive homeostatic mechanism

Previously, we demonstrated that *O*-GlcNAcylated chromatin-associated protein(s) are preferentially localized to the promoter region of a subset of genes in *C. elegans* (32) and in *Drosophila* (33). Following up on those observations, we now show that Pol II itself is dynamically GlcNAcylated *in vivo*, that the steady state distribution of *O*-GlcNAc and Pol II for most genes in wild type animals is unaffected by nutrient flux, that GlcNAcylated promoters are associated with high promoter proximal Pol II occupancy, and that loss of *O*-GlcNAc cycling results in profound changes in Pol II distribution in response to starvation and feeding. The relatively minor Pol II profile changes in fed vs. starved wild type *C. elegans* L1 larvae may initially seem counterintuitive. However, our results are consistent with those reported by Baugh et al. who assayed various starvation and feeding paradigms by ChIP-seq (19). These studies demonstrated that most genes do not show an acute response to nutrient flux, but instead maintain a steady state level of expression. Our current findings suggest that *O*-GlcNAc cycling underlies, at least in part, the near constant Pol II distribution genome-wide and the relatively minor transcriptional changes associated with dramatic changes in nutrient availability. In the absence of *O*-GlcNAc cycling, this homeostatic mechanism becomes deregulated and Pol II distribution across genes varies greatly in response to nutrient availability. Our results are consistent with a model in which dynamic *O*-GlcNAc cycling directly impacts Pol II distribution and dynamics for many, if not all, genes.

### The “CTD-code” in *C. elegans*

Although understudied in *C. elegans*, it is generally agreed that there are discrete steps of Pol II action during eukaryotic transcription cycles, including pre-initiation, initiation, pausing, elongation, and termination [reviewed in (3)]. These steps in the transcription process have been linked to specific modifications of the C-terminal domain (CTD) of the large subunit of Pol II that is comprised of numerous identical or variant repeats of the heptad amino acid sequence YSPTSPS. For example, transcriptional initiation and promoter-proximal pausing is associated with hyperphosphorylation of Ser-5 of the heptad repeats. This form of Pol II is converted to an elongating form by the action of PTEF-b, an enzymatic complex that phosphorylates Ser-2 of the heptad repeat and other negative regulatory substrates within the holoenzyme. A second Pol II pause occurs at the 3′ end of genes associated with termination and 3′ end processing. Relatively high levels of Ser-2, and low levels of Ser-5, phosphorylation are associated with this step of transcription. Thus, it is generally agreed that the distribution of CTD heptad repeat phospho-isoforms reflects the various functions of Pol II throughout the transcription cycle.

Profiling the distribution of several Pol II CTD phospho-isoforms with multiple antibodies failed to reveal the canonical pattern of eukaryotic transcription cycle events in *C. elegans* L1 chromatin preparations. Others came to a similar conclusion when examining profiles with several of the same Pol II antibodies we used (19, 20, 56). This suggests that either the mode of regulation for Pol II during the transcription cycle is different in *C. elegans* compared to other organisms or, more likely, that epitope recognition of the phosphorylated CTD *in vivo* is more complex than *in vitro*. We find that the overall pattern of distribution for multiple Pol II CTD phospho-epitopes reflects a shared characteristic of a particular set of selected genes that may reflect a shared mechanism of transcriptional regulation. For the 827 *O*-GlcNAc marked genes, one of the shared characteristics is enhanced promoter proximal Pol II occupancy. The 827 genes marked by *O*-GlcNAc at this developmental stage in *C. elegans* have been bioinformatically linked to longevity, stress and immunity (32). We have previously documented that the *O*-GlcNAc cycling mutants exhibit an altered stress response and have changes in longevity and insulin-dependent phenotypes (23, 24, 45). Moreover, these mutants have wholesale changes in steady state transcript levels suggesting that the observed alterations in Pol II profiles by ChIP are associated with transcriptional consequences.

### Transcriptional deregulation in *O*-GlcNAc cycling mutants is not restricted to the “*O*-GlcNAc marked” genes

In this study, we document the changes in gene expression in starved and fed worms using wild type (N2), *ogt-1* and *oga-1* mutant strains. The results of these studies suggest that while gene expression changes are widespread, the deregulated genes are not restricted to those 827 genes marked by *O*-GlcNAc at their promoters. We do not find a simple direct correlation between the presence of *O*-GlcNAc promoters and its deregulation of expressed in *O*-GlcNAc cycling mutants; these gene lists show only ~10% overlap, as reflected in the Venn diagrams shown in Supplemental Figure 6. The lack of statistically significant overlap in the gene lists is not surprising given that the magnitude of the profiles differences in the metagene analyses represent at most a ~40% change in Pol II occupancy and it is unclear that these changes alone would result in differences in steady state RNA levels. Moreover, gene expression in the highly invariant *C. elegans* developmental process is both cell type specific and temporally regulated. Many of O*-*GlcNAc-occupied promoters may be transcriptionally silent. *O*-GlcNAc marked promoters often reside between protein coding genes complicating assignment to a unique gene. In additional, posttranscriptional mechanisms and the influence of non-coding RNAs are difficult to predict using current tools. However, we can conclude that the presence of *O*-GlcNAc at a promoter of a given developmental stage is not necessarily predictive of transcriptional change upon loss of *O*-GlcNAc cycling.

### *C. elegans* pol II is dynamically *O*-GlcNAcylated *in vivo*

We find that Pol II is a substrate for GlcNAcylation in *C. elegans*, a has been reported previously in many different systems (33–38). Although the mammalian sites of GlcNAcylation include Ser and Thr residues of the CTD, the functional consequences of this modification have not previously been explored in a living organism. The large number of heptad repeats, each having up to four potential *O*-GlcNAcylation sites, suggests Pol II could be a predominant chromatin-associated protein detected by ChIP with antibodies specific for *O*-GlcNAc epitopes. This may explain, at least in part, the coincident profiles we observe for *O*-GlcNAc and Pol II at the 5′ end of many genes. Importantly, GlcNAcylation of the CTD residues that are also targets for phosphorylation would be expected to have profound effects on phosphorylation patterns and Pol II function. A complex interaction between CTD GlcNAcylation, phosphorylation, and transcription cycle progression might explain the many and diverse changes in Pol II patterns observed in *O*-GlcNAc cycling mutants. A complete analysis of this complex interplay will require a detailed analysis of the sites of phosphorylation and GlcNAcylation of the extended CTD domain of RNA Pol II. We note, however, that the redistribution of Pol II observed in *O*-GlcNAc cycling mutants was detectable using both phospho-independent and -dependent antibodies. This finding strongly suggests that *O*-GlcNAc is part of the complex “CTD-code” acting independently of known phosphorylation-dependent mechanisms.

### A model of *O*-GlcNAcylation and the “CTD-code”

**Figure 8:**
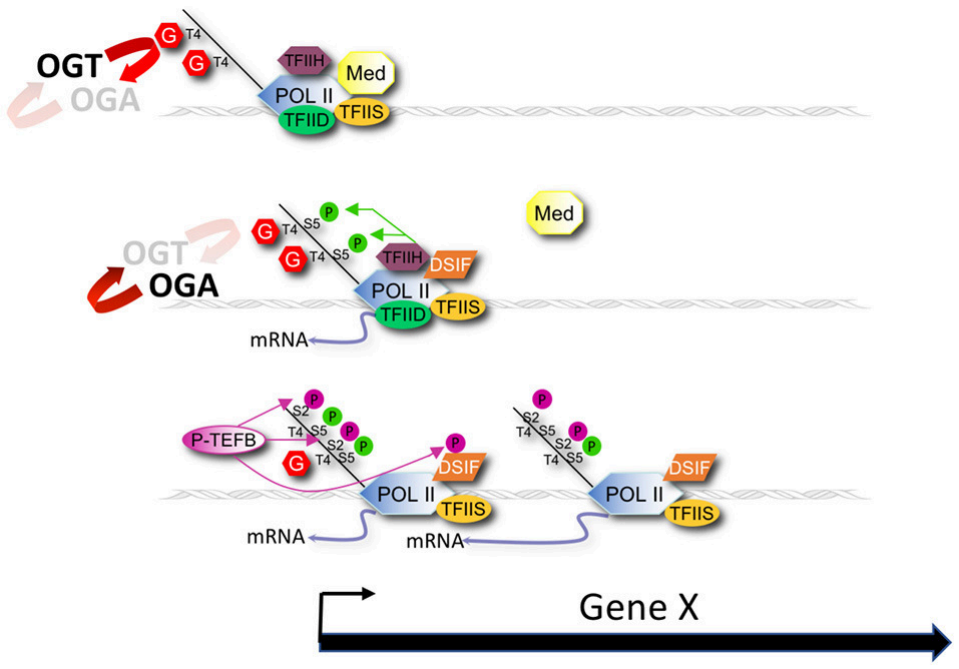
Modeling promoter region *O*-GlcNAcylation and Pol II dynamics. A model for the role of *O*-GlcNAc cycling in promoter proximal Pol II dynamics during nutrient flux based on our results is shown that also draws from earlier studies of Lewis and colleagues (37–40). OGT and OGA enzymatic activity dynamically cycles *O*-GlcNAc on Pol II, and other chromatin substrates, that localize at the promoter of many, if not all, genes. *O*-GlcNAcylation of the Pol II CTD competes with phosphorylation of Ser-2 and Ser-5 residues of the heptad repeats that are required for transcriptional initiation and elongation. Nutrition (e.g., fed condition) drives increased *O*-GlcNAcylation of Pol II that inhibits progression of the Pol II transcription cycle. Conversely, starvation decreases *O*-GlcNAcylation of Pol II, thus promoting transcription events. The consequence of the nutrient-sensitive *O*-GlcNAc cycle is a near constant, or buffered, level of promoter proximal Pol II. In the absence of *O*-GlcNAc activity (*ogt-1* mutant) this buffering system is lost, resulting in Pol II dis-regulation. In the presence of excess *O*-GlcNAcylation (*oga-1* mutant), the inhibitory effects of this modification on Pol II are only slightly greater than in wild type animals, thus minimally affecting Pol II dynamics.

A simple model extending from previous work (34–40), our current observations, and the role of CTD phosphorylation in transcription [reviewed in (3)] is that GlcNAcylation of the CTD (and perhaps other associated substrates) normally serves as a negative regulator of Pol II initiation or elongation by directly competing with phosphorylation events required for transcription cycle progression (Figure 8). This O*-*GlcNAc may serve to stabilize the CTD domain from degradation and serve to limit the action of kinases until the polymerase becomes promoter associated. In wild type animals, the potential increase in Pol II transcription driven by nutrient excess might be balanced by compensatory high levels of negatively acting GlcNAcylation, providing a self-adjusting regulator that would ensure near constant transcription of many genes during times of nutrient flux. In the *O*-GlcNAc cycling mutants, this transcriptional regulator either no longer exists (*ogt-1*) or is hyper-engaged (*oga-1*). The consequences of the deregulated *O*-GlcNAc system is a nutrient-dependent change in Pol II accumulation at the promoter region that could reflect changes in process associated with pre-initiation complex formation, transcriptional initiation, promoter proximal pausing, transcriptional elongation, or any combination thereof.

This model is derived from observations of the 827 most heavily *O*-GlcNAc marked genes, but it appears to be applicable to many genes. That is, promoter region levels of chromatin-associated GlcNAcylation fall on a continuum, which may reflect varying degrees of reliance on this mechanism of homeostatic control. For example, the Pol II profiles of the 827 GlcNAc-marked genes are extremely well buffered during nutrient flux in wild type animals. At the other extreme are those genes with very little promoter region GlcNAcylation and Pol II accumulation; instead, these genes have increasing Pol II signals toward the 3′ end of the gene. These presumably highly transcribed genes are nutrient unresponsive and constitutively on at very high levels. Another set of genes devoid of high levels of promoter GlcNAcylation is the one that responded dramatically to starvation. These genes, which are required for an acute transcriptional response to nutrient flux and/or stress, have high levels of Pol II occupancy at the promoter region but are, by definition, unbuffered.

There may be roles for promoter protein GlcNAcylation beyond the simple nutrient-responsive transcriptional regulation model we present. Animals lacking either one of the *O*-GlcNAc cycling enzymes, or both, are phenotypically wild type with respect to viability, fertility, and morphogenesis. Such observations suggest that there are nutrient regulated transcriptional responses that act in addition to, or that can supersede, *O*-GlcNAc-mediated controls. Moreover, many signaling pathways are integrated into, and impinge upon, transcriptional output, making the simple correlation of one chromatin modification (GlcNAcylation) with transcriptional regulation a difficult task. Like many metabolic regulatory networks, *O*-GlcNAc appears to function to fine-tune cellular responses in concert with many redundant and compensatory pathways. We find that this nutrient responsive, post-translational modification can profoundly influence Pol II occupancy across many genes and is, therefore, a novel player to consider in the context of gene regulation.

### *O*-GlcNAcylation and higher order chromatin structure

Regardless of the exact role of *O*-GlcNAc in chromatin regulation, this modification is specifically targeted to promoter regions. It is not yet clear how the enzymes of O-GlcNAc cycling are recruited to promoter regions and whether this recruitment is regulated. Targeting could be accomplished by site-specific complexes that recruit GlcNAcylated substrates or the *O*-GlcNAc cycling enzymes themselves. GlcNAcylation of nuclear pores has been extensively studied over the past 25 years (26, 62, 63), suggesting specific chromatin-associate domains within the nucleus could be linked to at least some of the *O*-GlcNAc promoter signals we observe. Another possible recruitment mechanism is suggested by the known mammalian interactions between the transcriptional repressor mSin3a and OGT (64). That is, specific interactions between *O*-GlcNAc cycling enzymes and promoter-specific regulatory, or Pol II-associated, factors might provide a specific docking site. Results from *Drosophila* demonstrate a related recruitment mechanism. Two groups have shown that Polycomb Response Elements (PREs) are prominent sites of *O*-GlcNAc modified chromatin and this mark is completely lost in null alleles of the *Drosophila sxc/ogt* gene (30, 31). PREs are the *cis*-acting elements that regulate transcriptional repression through Polycomb Regulatory Complexes (PRCs), as first uncovered in the regulation of HOX genes in the fly [reviewed in (65)]. Indeed, *sxc/ogt* genetically interacts with many Polycomb Group members and the PRC complex protein polyhomeiotic has been shown to be GlcNAcylated (30). We have yet to identify any genes that suggest GlcNAcylation of promoter regions for somatically expressed genes in *C. elegans* is involved directly in Polycomb-like repression. It is possible that *O*-GlcNAc-mediated braking of Pol II transcription observed in *C. elegans* has been co-opted to become part of a more robust transcriptional repression system in some species, such as for PRC function in flies and other organisms. It will be interesting to explore the potential for a similar role of GlcNAcylated chromatin in the *C. elegans* germline, where Polycomb-related factors are known to be involved in regulating global aspects of gene expression (66, 67).

### Biological significance of promoter region glcnacylation

The discovery of a dynamic, nutrient responsive mark that is localized to the promoter of many genes provides a direct link between cell metabolism and the transcriptional machinery. Such a link could have profound acute and long-term consequences on the transcriptional output of these genes. Our studies highlight the acute responses seen within 3 h of feeding, demonstrating that changes in nutrient status of cells can be reflected at specific gene promoters. It is not difficult to imagine scenarios in which prolonged exposure to feast or famine would similarly result in persistent epigenetic changes at specific promoters altering transcriptional patterns through many cell divisions somatically or across generations if functioning in the germline. For example, the persistent elevated serum glucose levels of diabetic mothers may reset the transcriptional state of genes within the developing fetus through an *O*-GlcNAc-mediated mechanism. Such a mechanism would provide a molecular explanation for the “vicious cycle” that describes the propensity of children of mothers with diabetes during pregnancy to develop obesity and diabetes at a young age (68). Other studies have linked paternal nutrition metabolic consequences in subsequent generations (69, 70). The challenge ahead is to fully understand the molecular consequences of *O*-GlcNAc cycling, both direct and indirect, on transcription and the relationship of this nutrient responsive epitope on the epigenetic control of gene expression.

## Experimental procedures

### Worm strains

Worm strains were cultured under standard conditions (71), unless otherwise noted. Strain used in this study were: wild type Bristol (N2), *ogt-1(ok430), ogt-1(ok1474)*, and *oga-1(ok1207)*.

### Starvation and feeding paradigm

Gravid adults were bleached, the embryos collected and hatched, and incubated in M9 buffer at 22°C with gentle shaking for 48 h. Half of this starved L1 population for each strain was collected and put on NGM plates with OP50 *E. coli* for 3 h at 22°C to serve as the fed treatment. Three hours was chosen because it was short enough to avoid major developmental changes induced by feeding that occur in the mid- to late-L1 stage and previous work demonstrated that transcriptional changes associated with feeding were near maximal by that time (19). Both starved and fed populations were collected, flash frozen, and stored at −70°C prior to further processing.

### Chromatin immunoprecipitation

Chromatin preparation from frozen samples and immunoprecipitations were carried out as previously described (32). The anti-*O*-GlcNAc antibodies used were RL2 [Abcam (ab2739); (72)] and HGAC85 [Thermo Scientific (MA1-076); (73)]; each gave similar results in all assays, although HGAC85 had better signal to noise for ChIP whereas RL2 was best for *O*-GlcNAc detection by Western blots. The four Pol II antibodies used were 8WG16 (Covance, MMS-126R) that was raised against wheat germ Pol II and recognizes both non-phosphorylated and phosphorylated isoforms of Pol II in *C. elegans* (19), ab5095 (Abcam) that was raised against the Ser-2-phospho (Ser-2-P) isoform of a consensus CTD diheptad repeat, a non-commercial anti-Ser-2-P CTD antibody (58), and anti-Ser-5-P antibody (57).

Immunoblots were performed as described previously (25).

### Gene model and genome-wide analyses

Sample preparation and genome-wide analyses of transcription and ChIP peaks as previously described (32); all array data is publicly available in GEO with accession numbers GSE40371 and GSE18132, respectively. To allow easy visualization of the ChIP-chip data from multiple genes simultaneously, a common gene model (metagene) of uniform length, extending from the translational start to stop codon was defined using genome version WS195 to match the arrays; non-array based gene definitions and analyses used WS235 and ModENCODE gene expression data was retrieved based on WS260. Each gene length was divided into ten equal segments and the signal intensity of all probes within each of these deciles was averaged. Flanking region distances for analysis were empirically determined by taking progressively longer flanks (1 kb increments) for the analysis; 3 kb of upstream sequence was determined to be optimal for data capture and this flank distance was applied to all ChIP data. The 3 Kb upstream flanking sequenced was divided into 20 bins and the probe intensities within each bin averaged and plotted along with the gene data as a moving average with five bins per step. Error bars at each point on the graph represent the standard error (standard deviation divided by the square root of the number of genes). For many plots, we calculated the ratio for the maximum value at the promoter region divided by the minimum value in the gene body region. The error values were calculated from the standard errors considering error propagation of this division. *T*-tests were performed using R, version 3.5.0. Bioinformatic analysis of transcriptional regulation was carried out using GEO2R and enrichment analysis was carried out using DAVID Bioinformatic Resources 6.8 (74, 75).

## Author contributions

MK and JH : Design, analysis and interpretation of data, Writing and editing. DL: Design, analysis and preparation of samples; SG and PW: Analysis and preparation of samples; SY: Biostatistical analysis of data; TF: Analysis, interpretation of data.

### Conflict of interest statement

The authors declare that the research was conducted in the absence of any commercial or financial relationships that could be construed as a potential conflict of interest.
