# Supplementary material for: Nutrient-Driven O-GlcNAcylation at Promoters Impacts Genome-Wide RNA Pol II Distribution
Source: Front Endocrinol (Lausanne). 2018 Sep 10;9:521. doi: 10.3389/fendo.2018.00521 (PMC6139338; doi:10.3389/fendo.2018.00521)
Supplement: Supplementary file 3 [file Presentation_1.PPTX]

## Slide 1
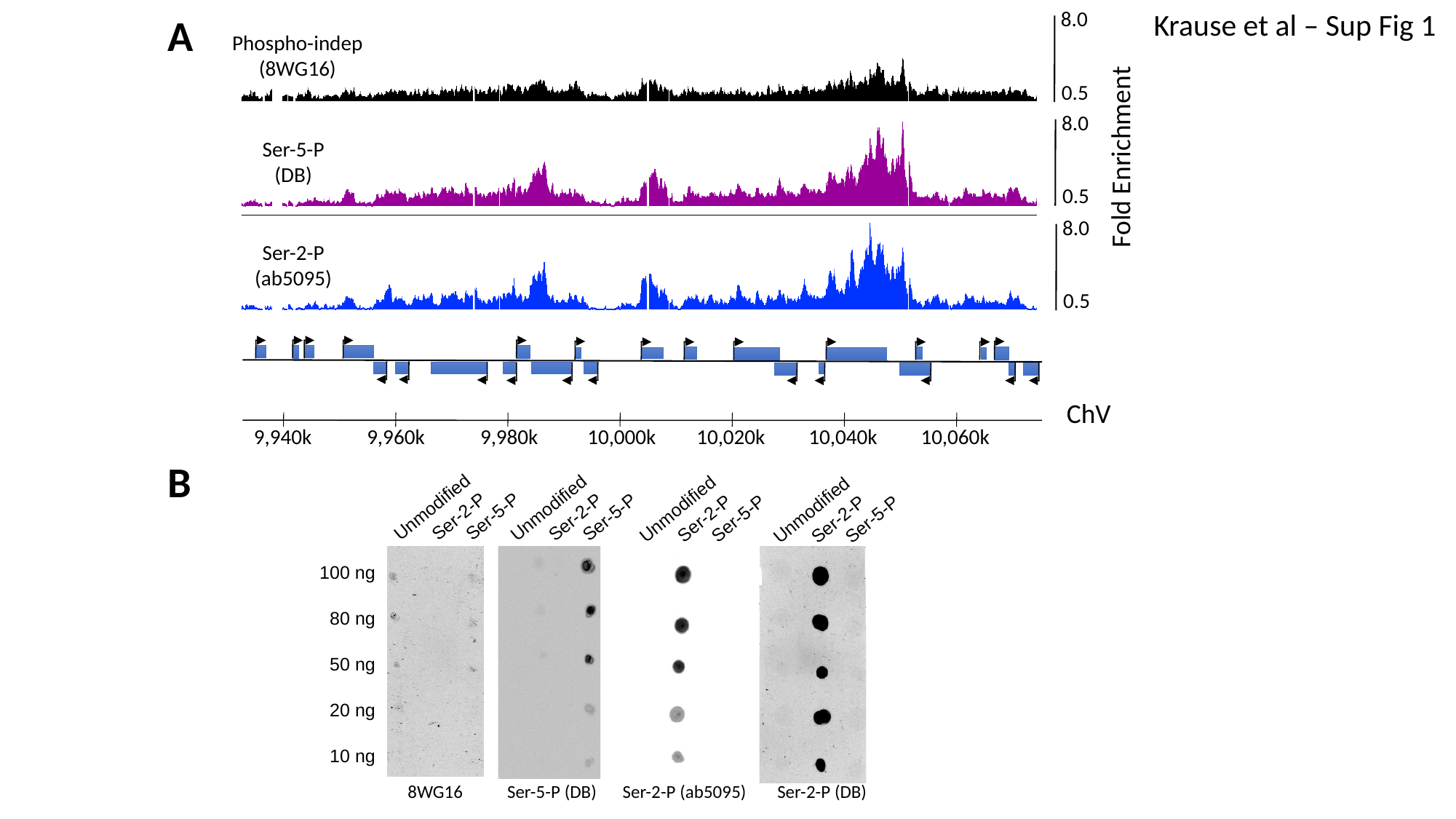

Krause et al – Sup Fig 1
8.0
0.5
A
Phospho-indep
(8WG16)
8.0
0.5
Ser-5-P
(DB)
Fold Enrichment
8.0
0.5
Ser-2-P
(ab5095)
ChV
9,940k
9,960k
9,980k
10,000k
10,020k
10,040k
10,060k
B
Unmodified
Ser-2-P
Ser-5-P
Unmodified
Ser-2-P
Ser-5-P
Unmodified
Ser-2-P
Ser-5-P
Unmodified
Ser-2-P
Ser-5-P
100 ng
80 ng
50 ng
20 ng
10 ng
8WG16
Ser-5-P (DB)
Ser-2-P (ab5095)
Ser-2-P (DB)

## Slide 2
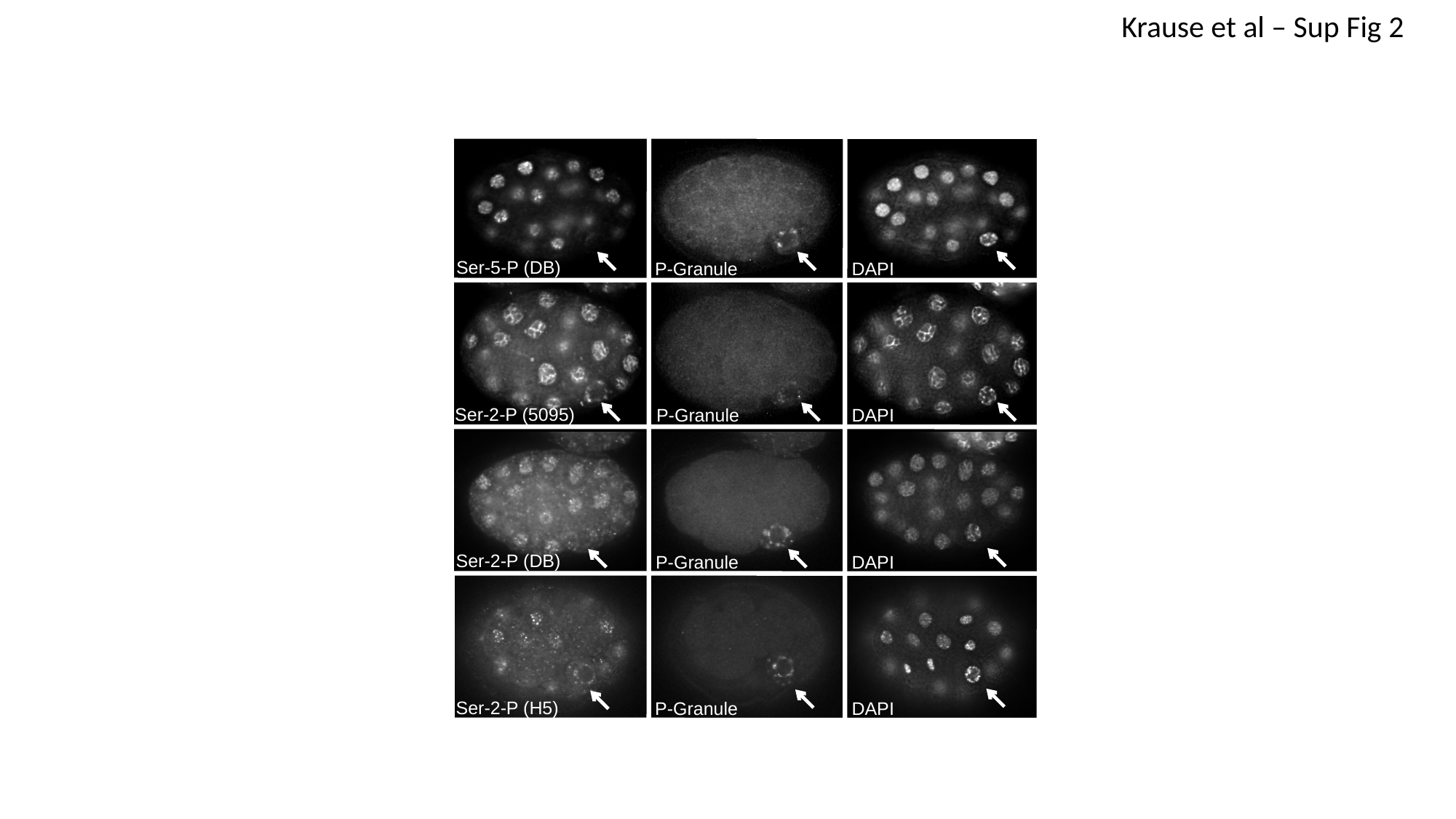

Krause et al – Sup Fig 2
Ser-5-P (DB)
P-Granule
DAPI
Ser-2-P (5095)
P-Granule
DAPI
Ser-2-P (DB)
P-Granule
DAPI
Ser-2-P (H5)
P-Granule
DAPI

## Slide 3
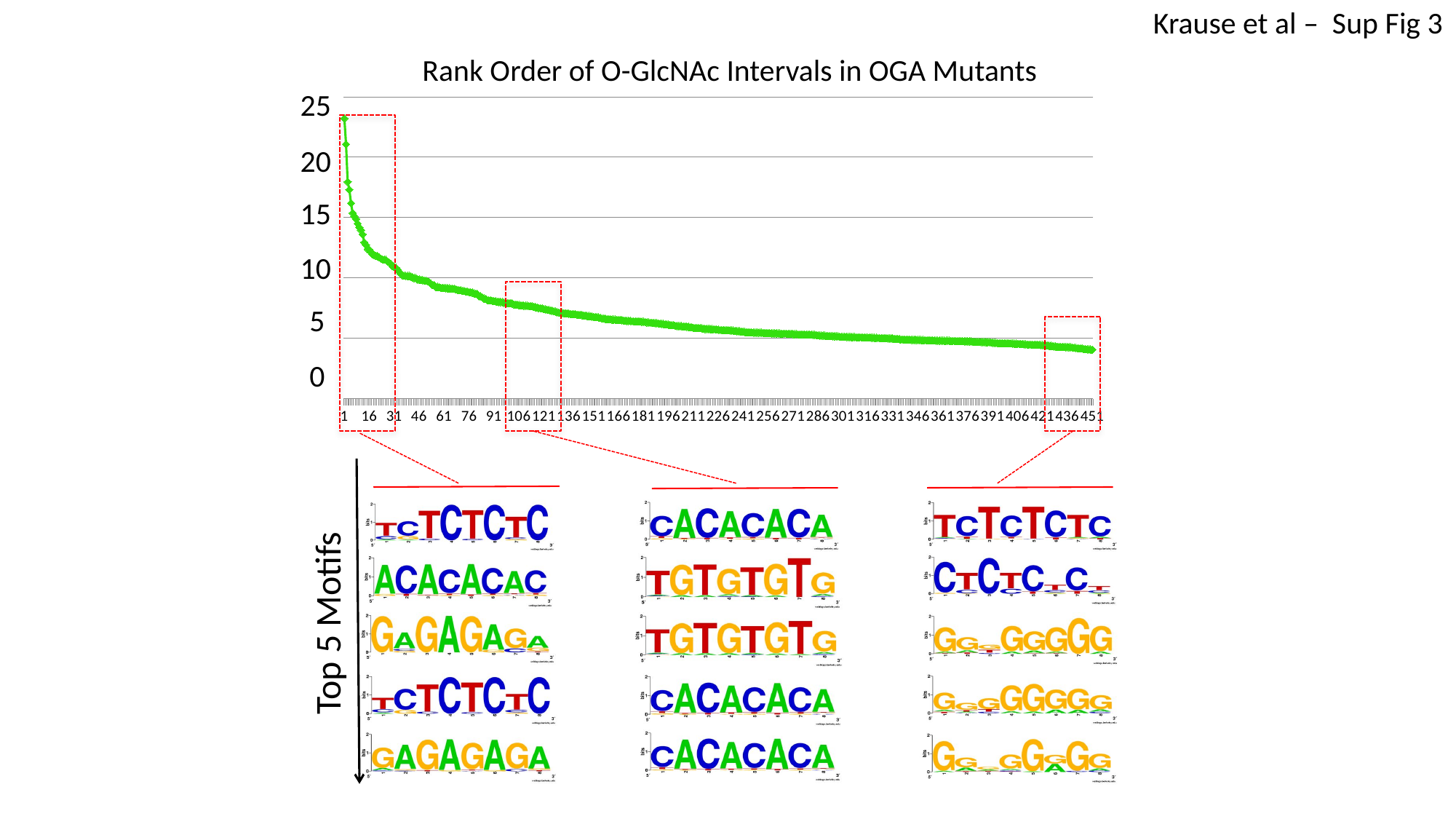

Krause et al – Sup Fig 3
Rank Order of O-GlcNAc Intervals in OGA Mutants
25
20
15
10
5
0
### Chart
| Category | Peak Val |
|---|---|
Top 5 Motifs

## Slide 4
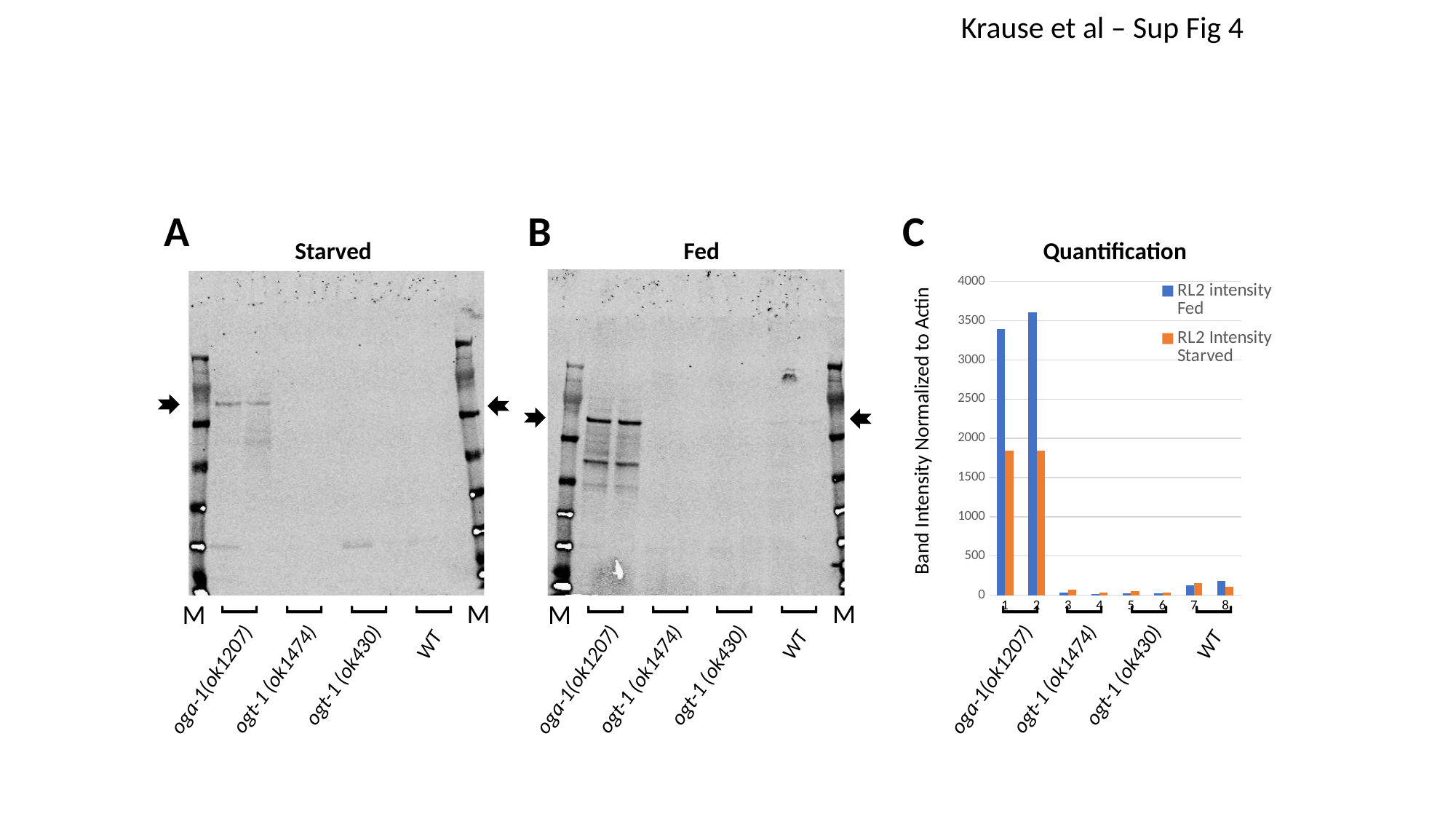

Krause et al – Sup Fig 4
A
B
C
### Chart
| Category | RL2 intensity Fed | RL2 Intensity Starved |
|---|---|---|Starved
Fed
Quantification
Band Intensity Normalized to Actin
]
]
]
]
]
]
]
]
M
M
WT
ogt-1 (ok430)
ogt-1 (ok1474)
oga-1(ok1207)
]
]
]
]
M
M
WT
ogt-1 (ok430)
ogt-1 (ok1474)
oga-1(ok1207)
WT
ogt-1 (ok430)
ogt-1 (ok1474)
oga-1(ok1207)

## Slide 5
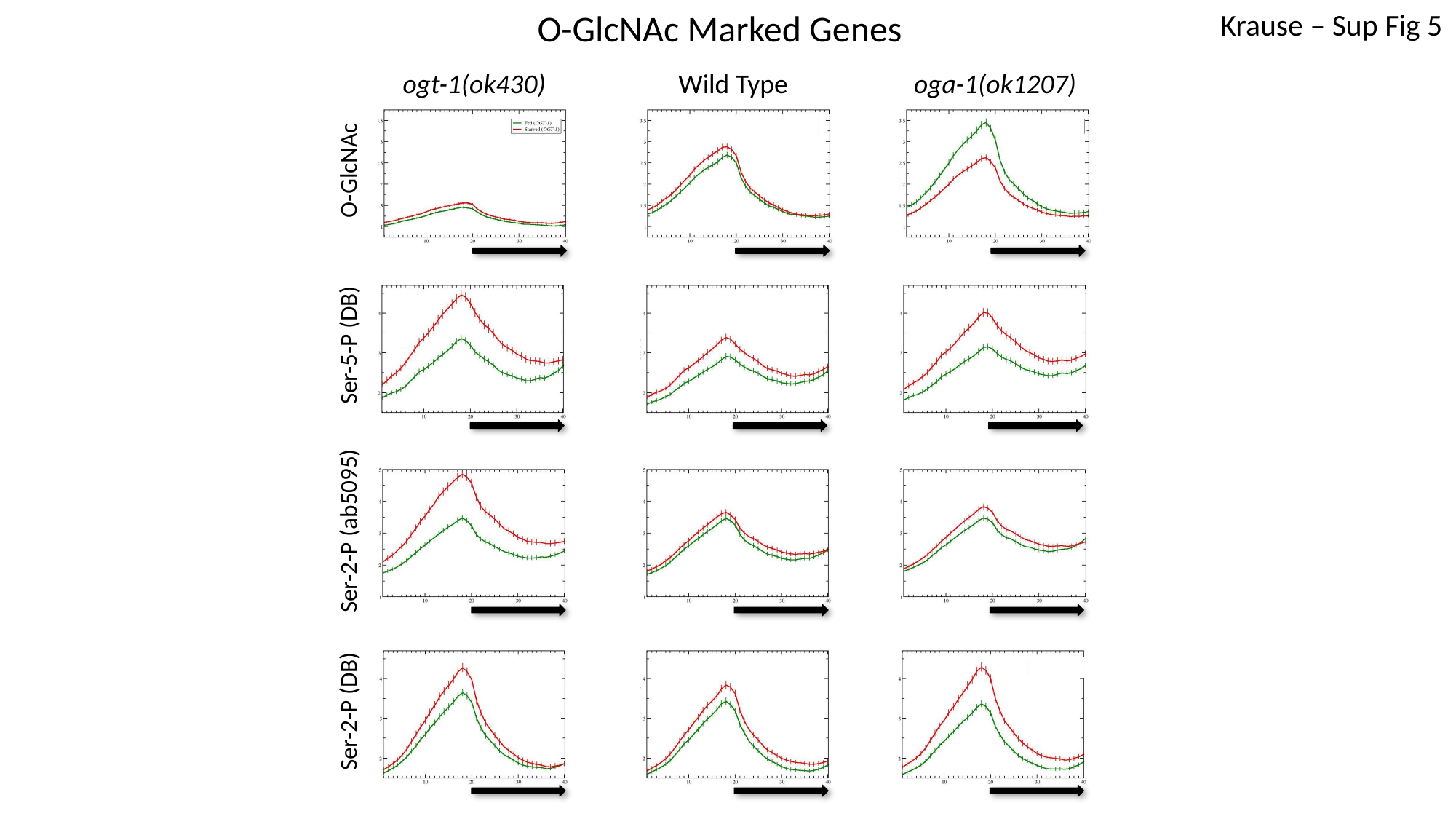

Krause – Sup Fig 5
O-GlcNAc Marked Genes
ogt-1(ok430)
oga-1(ok1207)
Wild Type
O-GlcNAc
Ser-5-P (DB)
Ser-2-P (ab5095)
Ser-2-P (DB)

## Slide 6
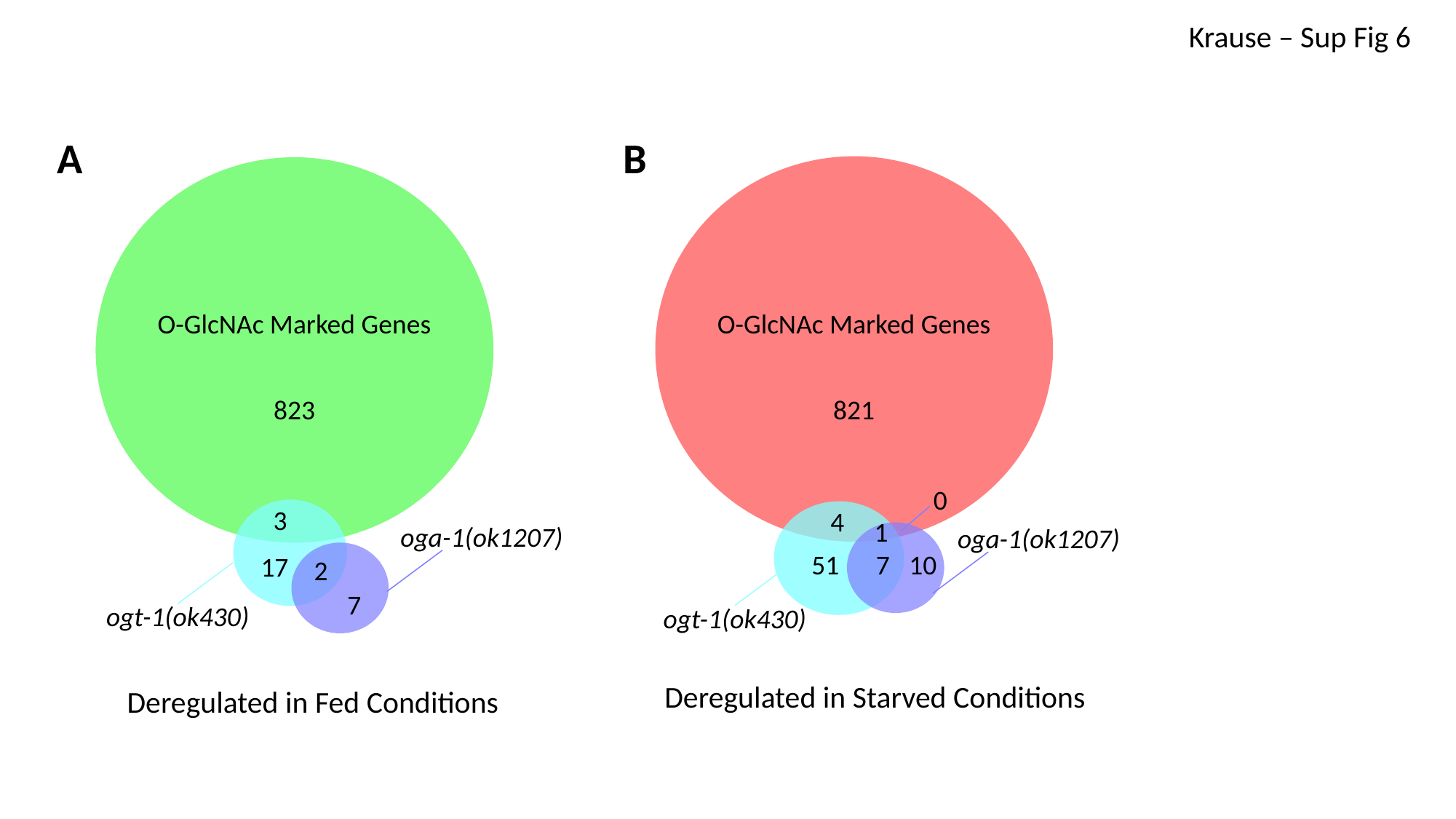

Krause – Sup Fig 6
A
B
O-GlcNAc Marked Genes
O-GlcNAc Marked Genes
823
821
0
3
4
1
oga-1(ok1207)
oga-1(ok1207)
51
7
10
17
2
7
ogt-1(ok430)
ogt-1(ok430)
Deregulated in Starved Conditions
Deregulated in Fed Conditions
