# Supplementary material for: Nutrient-Driven O-GlcNAcylation at Promoters Impacts Genome-Wide RNA Pol II Distribution
Source: Front Endocrinol (Lausanne). 2018 Sep 10;9:521. doi: 10.3389/fendo.2018.00521 (PMC6139338; doi:10.3389/fendo.2018.00521)
Supplement: Supplementary file 4 [file Data_Sheet_1.PDF]

Supplemental Figure Legends

Supplemental Figure 1: Multiple Pol II antibodies result in similar profiles by ChIP

(A) A comparison of the ChIP profile for several anti-Pol II antibodies that have different *in vitro* specificities for phospho-epitopes on the C-terminal domain (CTD). The signal intensity, uniformly scaled to fold-enrichment over input on the Y-axis, for a genomic region from ChV that includes multiple genes encoded on both strands (rectangles and arrows) is shown. Note that the pattern for the phospho-independent Pol II antibody (8WG16) is nearly identical to that of Ser-5-P (Bentley) and Ser-2-P (ab5095), with only the signal to noise ratio changing. (B) The specificity of all RNA Pol II antibodies used in this study was validated using a dot blot assay. Commercially available Pol II CTD heptad peptides (YSPTSPS) that were unmodified or phosphorylated in the Ser-2 or Ser-5 position were spotted in several different quantities (indicated to left) on nitrocellulose filter paper. Each spotted filter was then incubated with antibodies reported to recognize the Pol II CTD independent of phosphorylation status (8WG16), the Ser-5-P isoform, or the Ser-2-P isoform ab5095 and DB (Schroeder et al. 2000). All antibodies showed specificity to the reported epitopes in this assay.

Supplemental Figure 2: RNA Pol II antibodies validate by *in situ* staining of early *C. elegans* embryos.

Each phospho-specific RNA Pol II CTD antibody used for ChIP was tested *in vivo* by staining early *C. elegans* embryos and compared to controls. Under certain

fixation conditions (Seydoux and Dunn 1997; Batchelder et al. 1999) some Ser-2-P- and Ser-5-P-specific Pol II CTD antibodies have been shown to stain somatic cell, but not germline, nuclei; absence of staining in the germline reflects the relatively quiescent transcriptional state during early embryogenesis. As shown, both Ser-2-P- and Ser-5-P-specific antibodies detected somatic nuclei while failing to detect the germline precursor (P4; arrows) as marked by the P-granule antibody OIC1D4. DAPI staining of DNA is shown to identify all nuclei. By mid-embryogenesis, the germline precursors (Z2 and Z3) were strongly positive for all Pol II antibodies tested (data not shown). Pol II antibodies used were Ser-5-P from David Bentley (DB), Ser-2-P ab5095 (a), Ser-2-P from David Bentley (DB), and the control Pol II antibody H5 that has previously been reported (Seydoux and Dunn 1997).

Supplemental Figure 3: Rank ordered O-GlcNAc intervals and over-represented sequence motifs.

Top: 452 sequence intervals (X-axis) identified as O-GlcNAc associated in starved *oga-1* mutant L1 animals after O-GlcNAc (HGAC85) ChIP are plotted in rank order by their associated interval score (Y-axis). Bottom: For three groups of 30 intervals each (boxed in red), the top five over-represented sequence motifs identified by MDScan (Liu et al. 2002) are shown in descending order. All motifs are simple sequence repeats, albeit different sets of sequences emerge from different ranked groups.

Supplemental Figure 4: Nutrient-dependent changes in O-GlcNAc signals.

Total L1 animal protein samples from starved and fed populations of wild type (WT), *ogt-1(ok430), ogt-1(ok1474)* and *oga-1(ok1207)* separated by gel electrophoresis in paired lanes and assayed by Western blotting for O-GlcNAcylated peptides by staining with RL-2 (Abcam) antibody. Matched, synchronized L1 populations that were either starved (A) or fed for three hours (B) were compared, with quantification of the major bands for each lane determined (C); equal loading and transfer of protein samples was confirmed by actin staining and Ponceau S staining of the Western blots, respectively. Although O-GlcNAc signals in both wild type and *ogt-1* mutants were barely detectable, *oga-1(ok1207)* mutants had dramatically increased O-GlcNAc signals that were further elevated in fed conditions compared to starved.

Supplemental Figure 5: Nutrient-dependent changes in Pol II occupancy accompany loss of normal O-GlcNAc cycling.

The distribution of O-GlcNAc (HGAC85) and Pol II (three different antibodies) is shown for the previously identified 827 O-GlcNAc marked genes (Love et al. 2010). Profiles are shown for wild type and O-GlcNAc cycling mutants *ogt-1(ok430)* and *oga-1(ok1207)* relative to the metagene model (black arrow) in starved (red) and fed (green) conditions. Whereas wild type animals maintained near constant profiles of Pol II in the face of nutrient flux, both O-GlcNAc cycling mutants showed more dramatic changes in profiles in response to starvation or feeding for most Pol II antibodies used in these experiments. Data shown corresponds to Pol II antibodies for Ser-5-P from David Bentley (DB), Ser-2-P ab5095, and Ser-2-P from David Bentley (DB).

Supplemental Figure 6: Overlap of O-GlcNAc-Marked and Transcriptionally Deregulated Genes.

The Venn diagrams show the degree of overlap between the set of 827 O-GlcNAc-marked genes (Love et al. 2010) and those genes with a 2.8-fold, or greater, level of deregulated expression in *ogt-1(ok430)* and *oga-1(ok1207)* mutants compared to wild type in either fed (A) or starved (B) conditions. The degree of overlap between the gene sets fails to reach statistical significance (Fisher's Exact Test, $p<0.05$) with values for starved and fed deregulated mutant gene set overlaps equal to 0.12 and 0.06, respectively.

References

Batchelder C, Dunn MA, Choy B, Suh Y, Cassie C, Shim EY, Shin TH, Mello C, Seydoux G, Blackwell TK. 1999. Transcriptional repression by the Caenorhabditis elegans germ-line protein PIE-1. *Genes & development* **13**: 202-212.

Liu XS, Brutlag DL, Liu JS. 2002. An algorithm for finding protein-DNA binding sites with applications to chromatin-immunoprecipitation microarray experiments. *Nat Biotechnol* **20**: 835-839.

Love DC, Ghosh S, Mondoux MA, Fukushige T, Wang P, Wilson MA, Iser WB, Wolkow CA, Krause MW, Hanover JA. 2010. Dynamic O-GlcNAc cycling at promoters of Caenorhabditis elegans genes regulating longevity, stress, and immunity. *Proc Natl Acad Sci U S A* **107**: 7413-7418.

Schroeder SC, Schwer B, Shuman S, Bentley D. 2000. Dynamic association of capping enzymes with transcribing RNA polymerase II. *Genes & development* **14**: 2435-2440.

Seydoux G, Dunn MA. 1997. Transcriptionally repressed germ cells lack a subpopulation of phosphorylated RNA polymerase II in early embryos of Caenorhabditis elegans and Drosophila melanogaster. *Development* **124**: 2191-2201.
